# Impact of low- and high-molecular-mass components of human serum on NAMI-A binding to transferrin

**DOI:** 10.1007/s00775-015-1255-5

**Published:** 2015-03-20

**Authors:** K. Śpiewak, M. Brindell

**Affiliations:** Department of Inorganic Chemistry, Faculty of Chemistry, Jagiellonian University, Ingardena 3, 30-060 Kraków, Poland

**Keywords:** Transferrin, Albumin, Serum, NAMI-A, FPLC

## Abstract

Imidazolium *trans*-tetrachloridodimethylsulfoxideimidazolruthenate(III), NAMI-A, a novel antimetastatic ruthenium complex was investigated towards affinity to transferrin (Tf), whether Tf–Ru adducts might be formed after its intravenous injection. Studies were focused on the holotransferrin due to its preferential binding to transferrin receptor. Here, we showed that holotransferrin is able to bind NAMI-A as readily as apotransferrin. The simulation of biological conditions of human serum performed by application of simplified serum models allowed to analyse ruthenium distribution between transferrin and albumin. The presence of physiological concentration of albumin (ca. 18-fold excess over Tf) resulted in a twofold decrease of ruthenium binding to Tf. Interestingly, the introducing of low-molecular-mass components of serum dramatically increased the ruthenation of Tf. Intermolecular competition binding studies between transferrin and albumin showed that both proteins bound similar amount of ruthenium species. Investigation of NAMI-A binding to Tf in human serum showed that this protein was not the major binding partner for Ru complex. However, in spite of many competing proteins still the ruthenation of Tf was observed. The lack of free Ru species (protein unbounded) after incubation with human serum allowed to make an assumption of high affinity of NAMI-A towards serum proteins.

**Electronic supplementary material:**

The online version of this article (doi:10.1007/s00775-015-1255-5) contains supplementary material, which is available to authorized users.

## Introduction

**Fig. 1:**
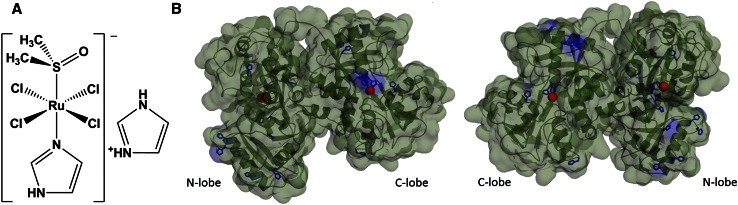
**a** Structure of NAMI-A. **b** Crystal structure of human serum holo-Tf showing the location of histidine residues (highlighted in *blue*) with solvent accessible surface, on the left and right front and back projection of Tf structure, respectively (PDB ID: 3QYT). This figure was made using PyMol [29]

Ru(III) complexes have promptly become an attractive alternative for the investigation of new metal-based anticancer agents [1–3]. One of the most promising drug candidates is (ImH)[*trans*-RuCl_4_(dmso)(Im)] (NAMI-A, Fig. 1a, where Im is imidazole), which successfully completed phase I clinical trial [4]. Interestingly, in most of in vitro and in vivo studies, NAMI-A was inactive as cytotoxic agent, however, exhibited a pronounced antimetastatic activity [5–8]. Moreover, very recent findings suggest that NAMI-A is not internalized into cells and consequently, it acts at an extracellular level [9]. Lately, quite surprisingly, the high cytotoxicity of NAMI-A against a few leukaemia cells was demonstrated but still the uptake of Ru complex was marginal and the inhibition of KCa 3.1 channels was proposed as a mode of action [10]. In view of these outcomes, the hypothesis that NAMI-A can be transported via transferrin receptor-mediated endocytosis pathway, proposed by many researchers [11–15] seems to be questionable. Though another hypothesis can be proposed that ruthenated transferrin can block the transferrin receptor (TfR) and by this can decrease the iron transport into cell. This hypothesis has never been closely inspected. The fundamental enquiry regards evaluation of the ability of NAMI-A to bind to transferrin (Tf) as a natural consequence of intravenous injection, despite a huge excess of albumin in serum (HSA). Typically, in healthy individuals, the concentration of HSA is ca. 0.6 mM [16, 17] while Tf varies between 25 and 50 µM [18].

The Tf is a monomeric glycoprotein (80 kDa) consisted of two lobes: N- and C-lobe connected by a short peptide linker (Fig. 1b). Tf binds two Fe^3+^ ions in association with the binding of an anion, mostly bicarbonate. Each lobe binds iron tightly but reversibly with association constant ca. 10^22^ M^−1^ [19]. Both iron-binding sites are identical (two tyrosines, a histidine and an aspartic acid). Only 30 % of Tf exists as iron-saturated diferric form of Tf (holo-Tf) [18]. Holo-Tf binds to the TfR located on the outer cell membrane with a high affinity (nanomolar dissociation constant) at neutral pH (~7.4) [20], while the iron-free form of Tf (apo-Tf) has a marginal affinity to TfR [21]. So far, most of the studies have focused on the interaction between NAMI-A and apo-Tf as a potential drug transporter [22–24]; however, our approach concentrates on holo-Tf due to high affinity of iron-saturated form to TfR.

In this study, several issues were addressed: (1) the influence of the iron-occupied binding site on the overall association constant and Ru content in ruthenated Tf, (2) the competition in NAMI-A binding to Tf vs. HSA, and finally (3) the effect of low- and high-molecular-mass constituents of human serum on NAMI-A binding to Tf. The investigation was carried out with the use of various serum models as well as human serum. A special care was undertaken to use freshly prepared aqua solutions of NAMI-A to prevent its advanced hydrolysis undergoing at pH 7.4 (dissociation of chlorido and dmso ligands) [25–28]. To determine the percentage of Ru content in proteins, the protein-unbounded Ru as well as ruthenated Tf and HSA were separated using fast protein liquid chromatography (FPLC). The ruthenium content was determined for the mineralized samples by the inductively coupled plasma mass spectrometry (ICP-MS), while the protein concentration using the enzyme-linked immunosorbent assay (ELISA) specific for each protein.

## Experimental

### Chemicals

NAMI-A, (HIm)[*trans*-RuCl_4_(DMSO)(Im)] was prepared according to the previously reported procedure [30]. Its purity was checked by the elementary analysis. The analysis calculated for NAMI-A (*M* = 458.17): C 20.95; H 3.30, N 12.23, S 6.99, found: C 21.37, H 3.33, N 12.03, S 6.90. Human apo-Tf and holo-Tf (powder, BioReagent, suitable for cell culture, ≥98 %), HSA (powder, fatty acids free, globulin free, ≥99 %) were obtained from Sigma-Aldrich (Germany). All used chemicals were obtained in the highest available purity from Sigma-Aldrich, POCH S.A. (Gliwice, Poland), Alfa Aesar (Massachusetts, USA) and Merck Millipore. Control human serum and normal level BioNorm were purchased from BioMaxima (Lublin, Poland). Human Tf ELISA (immunoperoxidase assay for determination of transferrin in sera) and HSA ELISA (immunoperoxidase assay for determination of albumin) were obtained from ICL (Portland, USA). All solutions were prepared in MilliQ quality water, and eluents were filtered through 0.45 μm membrane filters and degassed.

### Samples preparation

The NAMI-A stock solutions were prepared by dissolving NAMI-A powder in MilliQ water and its concentration was determined spectrophotometrically using molar absorptivity at 390 nm of 3644 M^−1^ cm^−1^ as it was described previously [31]. All protein stock solutions were prepared by dissolving in an appropriate buffer and concentration was measured spectrophotometrically using absorption coefficients at 280 nm of 86,400, 84,000 and 42,000 M^−1^ cm^−1^ for holo-, apo-Tf and HSA, respectively [32, 33]. In most of the experiments, the concentration of NAMI-A was kept constant (0.70 mM) and it corresponds to the typical Ru concentration in the blood of patients within 24 h after treatment (0.4–0.8 mM Ru) [34]. Unless stated otherwise, the NAMI-A was kept at 20-fold excess over Tf. Serum Models 0 and 0* were prepared by mixing of proteins dissolved in 50 mM TRIS/HCl buffer pH 7.4 containing 0.1 M NaCl and 25 mM NaHCO_3_ at desired ratio (Fig. 4). Serum Models 1 and 2 were made by mixing components listed in Fig. 4 followed by pH adjusting to 7.4 and the addition of an appropriated amount of proteins. NAMI-A was added into all these models or into human serum by the addition of small volume of concentrated stock solution, usually 2–5 μl. Samples with Ru complex were incubated 24 h at 37 °C. Details concerning intermolecular competition studies are described in the caption of Table 1.

**Table 1:** Ru fraction [mol/mol(protein)] in ruthenated Tf or HSA produced after (1) incubation of NAMI-A, Tf and HSA at 1:1:1 molar ratio for 24 h in buffer; (2) incubation of HSA with 20 molar excess of NAMI-A, followed by purification by FPLC separation from free Ru species, and then mixed with Tf at 1:2 molar ratio

	Model	Molar ratio	Ru/Tf	Ru/HSA
(1)	holo-Tf/HSA/NAMI-A	1:1:1	0.39 ± 0.08	0.65 ± 0.07
	apo-Tf/HSA/NAMI	1:1:1	0.24 ± 0.09	0.56 ± 0.06
(2)	(HSA/NAMI)/holo-Tf	(1:1):2	0.31 ± 0.02	0.67 ± 0.03
	(HSA/NAMI)/apo-Tf	(1:1):2	0.23 ± 0.02	0.71 ± 0.05

Buffer conditions: 50 mM TRIS/HCl, 100 mM NaCl, 25 mM NaHCO_3_, pH 7.4, 37 °C

### Spectrofluorimetric titration

Fluorescence measurements were performed on a spectrofluorimeter PerkinElmer LS55 equipped with the circulation flow PolyScience thermostat in a quartz cell with a 1-cm path length. The emission spectra were recorded between 305 and 500 nm upon excitation at 295 nm. The average of three scans was subjected to smoothing and the fluorescence intensities were corrected due to dilution effects as previously described [22]. The fluorescence lifetime measurements were performed with a single photon counting technique using Fluorolog-3, Horiba JobinYvon. The excitation wavelength was set at 265 nm (NanoLed Diodes) and the lifetime of fluorescence was monitored at 332 nm. Details are described in Ref. [22].

### Separation of protein fractions

FPLC was applied to separate the mixture comprising the unbound ruthenium species, Tf–Ru adducts and HSA–Ru adducts. The chromatographic system ÄKTA Purifier 10 and ÄKTA Pure (GE Pharmacia) with injection loop of 100 μl or 1000 μl and fraction collector FRAC-901 were used for separation. The separation was monitored spectrophotometrically at 280 nm (Monitor UV-900, GE Pharmacia) as well as by measuring the conductivity (Monitor pH/C-900, GE Pharmacia). Anion exchanged MonoQ 5/50 GL column (Tricorn, GE Healthcare Life Science; 5 × 50 mm I.D., 10 μm particle diameter) was applied to the separation of ruthenated proteins. Solutions of 0.02 M BIS–TRIS/HCl pH 7.0, A, and 0.02 M BIS–TRIS/HCl, 1 M NaCl pH 7.0, B, were used as eluents. All samples were filtered through 0.22 μm syringe filter before column application. Separation conditions were used as follows: injection volume: 100 μl, target concentration of B: 50 %, gradient length: 20 column volumes (CV), flow rate: 1 ml/min. Afterwards, the separation column was washed with 5 CV of eluent B and re-equilibrated with eluent A, 5 CV. Alternatively, the excess of Ru species was removed by membrane dialysis against 50 mM TRIS/HCl buffer pH 7.4 or water.

CaptureSelect™ Transferrin Affinity Matrix was used for a one-step transferrin purification from human serum. An empty column was packed with 2.5 ml of these resins. The column was equilibrated with 10 CV of the equilibration buffer: PBS, pH 7.4. Serum was filtered through 0.22 μm syringe filter and 400 μl of sample was injected into the column. Unbound serum components were washed with 5 CV of equilibration buffer. Transferrin was eluted with 5 CV of the elution buffer: 20 mM Tris, 50 % (v/v) propylene glycol, 1.0 M NaCl, pH 7.4 (chromatogram is presented in Fig. S1).

The abundance of proteins in human serum and after transferrin isolation by affinity chromatography was examined using 12.5 % polyacrylamide gel electrophoresis under denaturing conditions (SDS-PAGE) [35]. Gels were stained with Coomassie Brilliant Blue and documented using GelDoc-It™ 310 Imaging System (Upland, CA, USA).

Alternatively, the separation of protein fraction from low-molecular-mass components of human serum after incubation with NAMI-A was performed by ultrafiltration through a 3-kDa cutoff filter.

### Determination of protein and ruthenium concentration

The Tf and HSA concentrations in the studied systems were determined using human Tf or HSA ELISA kits, respectively. Standard curves were prepared with the supplied standards with concentration ranging from 9.375 to 600 ng/mL for Tf and from 6.25 to 200 ng/mL for HSA. The tested samples were diluted appropriately in a sample diluent buffer and the tests were performed according to the manufacturer’s protocol. ELISA 96-well plates were read using Tecan Infinite 200 Reader plate at 450 nm for both Tf and HSA. The total protein concentration in human plasma was measured by Bradford method.

The total Ru content in samples was measured by the application of ICP-MS using ELAN 6100 Perkin Elmer spectrometer. Prior to the determination of Ru content, 100 μL of the samples was mineralized using 500 μl of Ultrapure concentrated nitric acid and then diluted with water.

The experiments were carried out at least in triplicate, unless stated otherwise.

## Results and discussion

### Affinity of NAMI-A towards holo-Tf

**Fig. 2:**
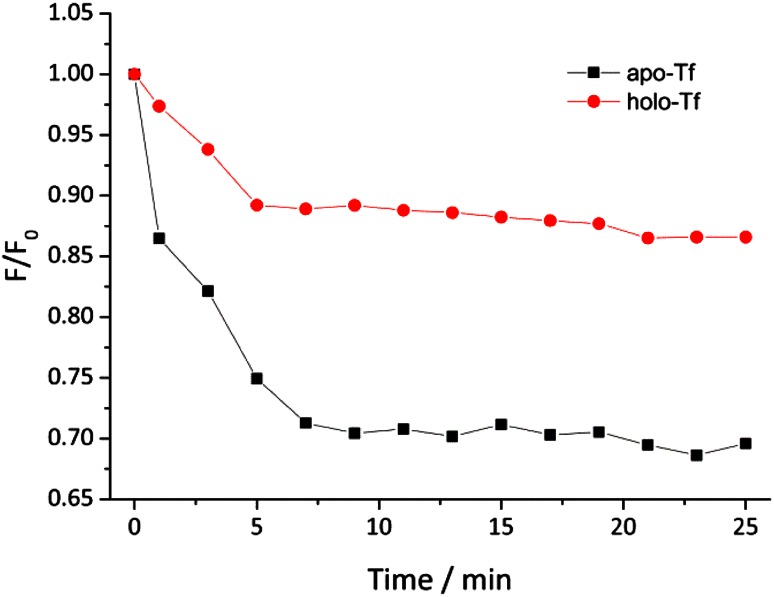
Kinetics of holo-Tf fluorescence quenching by NAMI-A. Experimental conditions: [holo-Tf] = 2.0 μM, [NAMI-A] = 40 μM; Tris/HCl pH 7.4; [NaCl] = 0.1 M; [NaHCO_3_] = 25 mM, *T* = 37 °C; *λ*_ex_ = 295 nm; *λ*_em_ = 338 nm

The quenching of intrinsic protein fluorescence upon ligand binding is a relatively quick and easy method for studying this interaction. The indole groups of tryptophan residues are the dominant source of UV absorbance and emission in proteins and its emission is sensitive to changes in the local microenvironment and can be used to monitor protein–ligand interactions [36]. Upon addition of NAMI-A to holo-Tf solution, the intensity of protein fluorescence gradually decreased and the reaction was completed within 5 min (see Fig. 2). The reaction time was similar to that measured for apo-Tf; however, the relative fluorescence intensity of holo-Tf–NAMI-A adduct is much higher than for apo-Tf–NAMI-A. Taking into account that holo-Tf is ‘closed’ iron-bound form, His and Trp residues are less accessible for NAMI-A so that the quenching is lower in comparison with apo-Tf.

**Fig. 3:**
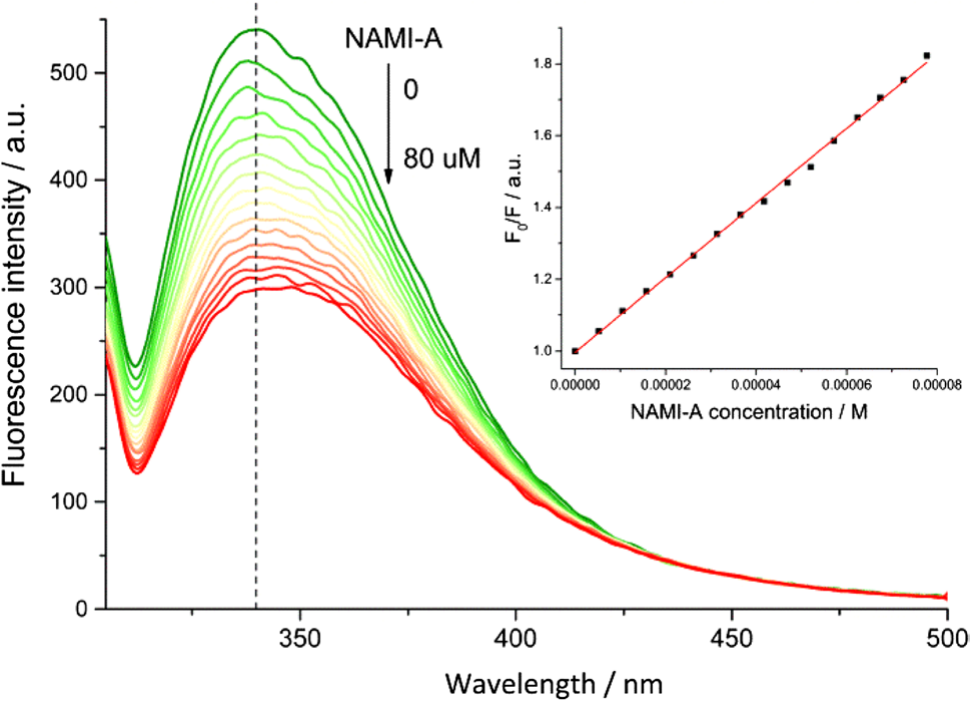
Fluorescence emission spectra for holo-Tf in the presence of increasing concentration of NAMI-A. *Inset* Stern–Volmer plot showing the influence of the increasing concentration of NAMI-A on the fluorescence intensity of holo-Tf. Experimental conditions: [holo-Tf] = 2.0 μM, [NAMI-A] = 0–77 μM; Tris/HCl pH 7.4; [NaCl] = 0.1 M; [NaHCO_3_] = 25 mM; *T* = 37 °C *λ*_ex_ = 295 nm; *λ*_em_ = 338 nm

A decrease in holo-Tf florescence was observed during NAMI-A addition in a concentration-dependent manner (Fig. 3). Ru complex addition caused the maximum emission shift towards longer wavelength from 338 to 346 nm. The observed red shift implies the formation of holo-Tf–Ru complex adduct that has more polar microenvironment in the vicinity of tryptophan residues. In apo-Tf, where Fe(III) ions are not present in binding sites the maximum of fluorescence is at 332 nm while NAMI-A addition induced a shift to 338 nm [22]. The observed spectral changes suggest that iron ion is not released upon NAMI-A complex binding, that was confirmed by ICP-MS measurements.

Fluorescence quenching data were analysed according to the Stern–Volmer equation [36]:

1 $$\frac{{F_{0} }}{F} = 1 + k_{\text{q}} \tau_{0} \cdot\left[ Q \right] = 1 + K_{\text{SV}} \cdot[Q],$$

where *F*_0_ and *F* are the fluorescence intensities of holo-Tf in the absence and presence of quencher (*Q*, NAMI-A), respectively; *k*_q_ is the bimolecular quenching constant; *τ*_0_ is the average lifetime of the holo-Tf fluorescence in the absence of quencher, [*Q*] is the concentration of quencher, *K*_SV_ is the Stern–Volmer constant. The plot of *F*_0_/*F* versus [*Q*] was linear (compare Fig. 3 inset) up to almost 40-fold excess of Ru complex over holo-Tf. Based on Eq. (1), the calculated value of *K*_SV_ was found to be 10 300 ± 100 M^−1^ at 37 °C. The measured value of the average lifetime of the holo-Tf in the absence of any quencher was ca. 2 × 10^−9^ s^−1^; therefore the calculated *k*_q_ equals 5 × 10^12^ M^−1^ s^−1^. Such high value of the bimolecular quenching constant points out the static quenching and allows for consideration of the Stern–Volmer constant as an association constant for the formation of holo-Tf–Ru complex(es) adducts [22, 37].The association constant for holo-Tf is slightly lower than for apo-Tf (12 800 ± 300 M^−1^) [22]. A moderate NAMI-A association constant for binding to holo-Tf can promote the release of Ru complex in cellular environment (e.g., at lower pH in lysosome) better than its liberation from apo-Tf–NAMI-A adduct. The obtained results indicate that Ru can bind not only to iron-binding sites like in apo-Tf, but also to other non-specific sites. Up to now, only X-ray structural studies on human lactoferrin (its structure closely matches that of human Tf) soaked with KP1019 (indazolium *trans*-[tetrachlorobis(1H-indazole)ruthenate(III)]) are available in the literature and they showed the presence of two types of binding sites: a high affinity in N-lobe iron-binding cleft and lower affinity sites at surface exposed His residues [38]. They pointed out that His residues are important in Ru complexation. Tf possesses 17 His residues, many of which are exposed to the protein surface and accessible for solvent molecules, and are thus potential ruthenium-binding sites (Fig. 1b). On the other hand, very recent in silico studies have shown that aquo-ethylenediaminetetraacetatoruthenium(III) forms hydrogen bonds upon binding to apotransferrin [39].

### Holo-Tf-bound Ru fraction

Holo-Tf was dissolved in buffer (50 mM Tris/HCl, 100 mM NaCl, 25 mM NaHCO_3_, pH 7.4) and incubated with 20-fold excess of freshly prepared NAMI-A for 24 h at 37 °C, then injected into anion exchange MonoQ 5/50 GL column for separation of free Ru species from holo-Tf-bound one (Fig. S2). Ru content in holo-Tf-bound fraction was quantified using ICP-MS method, while holo-Tf concentration was determined using ELISA. The Ru fraction [mol/mol(holo-Tf)] in ruthenated holo-Tf adducts was found to be 1.3 ± 0.1. It can be presumed that holo-Tf is able to bind at least one Ru ion, probably with moderate affinity; however, the binding of additional Ru ion with lower affinity cannot be excluded. Moreover, the presented data proved that ruthenated holo-Tf adduct was stable enough to be separated from free Ru species, suggesting the irreversible type of interaction under employed experimental conditions. This assumption was also supported by the determination of similar holo-Tf-bound Ru fraction after 24 h of membrane dialysis against either buffer or water. Our measurements using ICP-MS method as well as previously published data [40, 41] indicate that Ru ion does not replace iron in binding sites. Interestingly, even though the binding site in holo-Tf is occupied by iron, still holo-Tf offers better environment for binding of NAMI-A, which in case of apo-Tf was found to be at last two times lower (<0.6) [22]. This can arise from substantial structural differences between apo and holo forms of Tf, which, in turn, influence on His residue accessibility for Ru binding.

### Intermolecular competition studies: Tf versus HSA

NAMI-A affinity to both Tf and HSA was checked by analysis of Ru distribution between these proteins. To this end, two types of experiments were performed. Tf, HSA and NAMI-A were mixed at 1:1:1 molar ratio and incubated for 24 h in buffer at 37 °C. Then the mixture was separated on the MonoQ column (see Fig. S3), protein fractions were collected and Ru content per proteins was measured using ICP-MS and ELISA techniques. While NAMI-A interacted with HSA and Tf at the same concentration, a slightly higher amount of Ru was bound to HSA than to Tf (see Table 1). The NAMI-A complex had a slightly lower affinity towards apo- versus holo-Tf, and also more protein-unbound Ru species remained in solution containing a mixture of HSA with apo-Tf. This further supports assumption that NAMI-A complex binds to holo-Tf with higher extent.

**Fig. 4:**
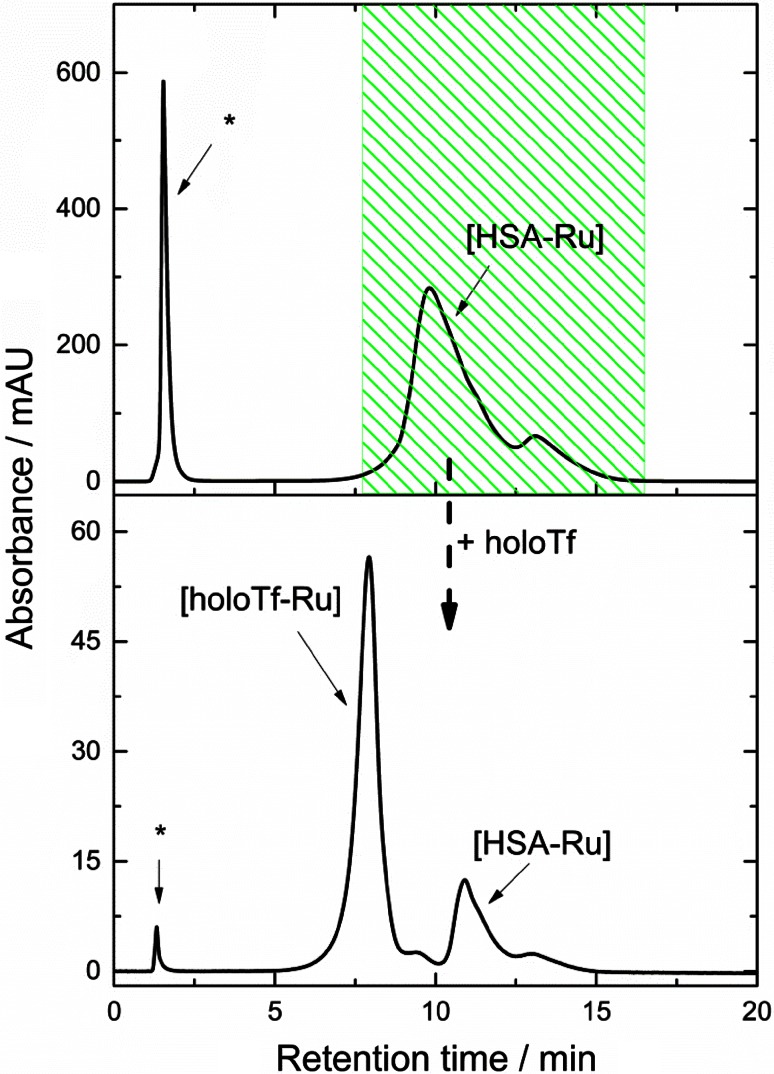
**a** Purification of [HSA–Ru] adduct from a mixture of HSA and NAMI-A incubated at 1:1 molar ratio for 24 h in buffer. **b** Separation of ruthenated proteins (holo-Tf and HSA) after addition to [HSA–Ru] adduct holo-Tf at 1:2 molar ratio and incubation for 24 h in buffer. *Asterisk* denotes the peak from solvent front comprising unbound Ru species. Buffer conditions: 50 mM TRIS/HCl, 100 mM NaCl, 25 mM NaHCO_3_, pH 7.4; 37 °C

Alternatively, NAMI-A was incubated with HSA at molar ratio 20:1 for 24 h at 37 °C, and then the HSA–Ru adduct was separated from free Ru species on the MonoQ column (see Fig. 4a). To the purified HSA–Ru adduct fraction, two molar excess of Tf over HSA was added and the mixture was incubated for 24 h at 37 °C. Subsequently, the ruthenated proteins were separated (see Fig. 4b) and Ru fraction in both types of adducts was assessed and the results are summarized in Table 1. The analysis of ruthenium distribution shows that both apo- and holo-Tf are able to sequester Ru ions from HSA, though albumin is still the major location of Ru ions. It was proposed for Pt-based drugs that platinated HSA can act as a Pt storage system [42]. One can assume by analogy that ruthenated HSA can serve as a reservoir of Ru ion for Tf in case of NAMI-A complex.

### Ru distribution in serum models

**Fig. 5:**
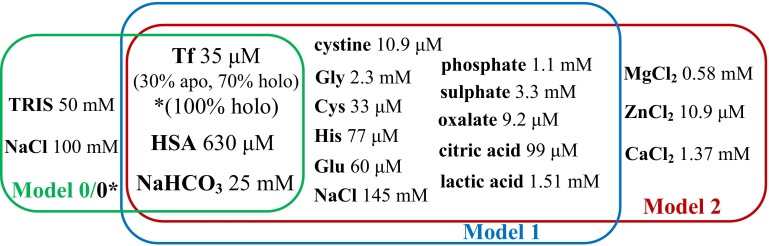
Serum models’ compositions (pH 7.4)

To achieve a better insight into NAMI-A behaviour in biological environment, Ru distribution between Tf and HSA was analysed in three types of serum models whose composition is presented in Fig. 5. The simplest Model 0 comprised the physiological mixture of Tf (30 % of holo and 70 % of apo forms [18]) and HSA at concentrations typically found in human serum dissolved in physiological type buffer (pH 7.4). It was used to clarify the impact of high HSA excess on Ru binding to Tf. For comparison, the Model 0*, possessing Tf only fully saturated with iron was also included. Serum Models 1 and 2 were prepared based on the composition proposed by Harris [43] and used by other researchers [44, 45] with slight modifications (see Fig. 5). Model 1 was composed of amino acids with (Cys, His, Glu) or without (Gly, cysteine) additional coordinating group, inorganic (chloride, carbonate, phosphate, sulphate) and organic (oxalate) sodium salts, lactic and citric acids, as well as the same content of proteins like in Model 0, all adjusted to pH 7.4. Model 2 had the same components as 1, however, was enriched with metal ions: calcium, magnesium and zinc.

**Fig. 6:**
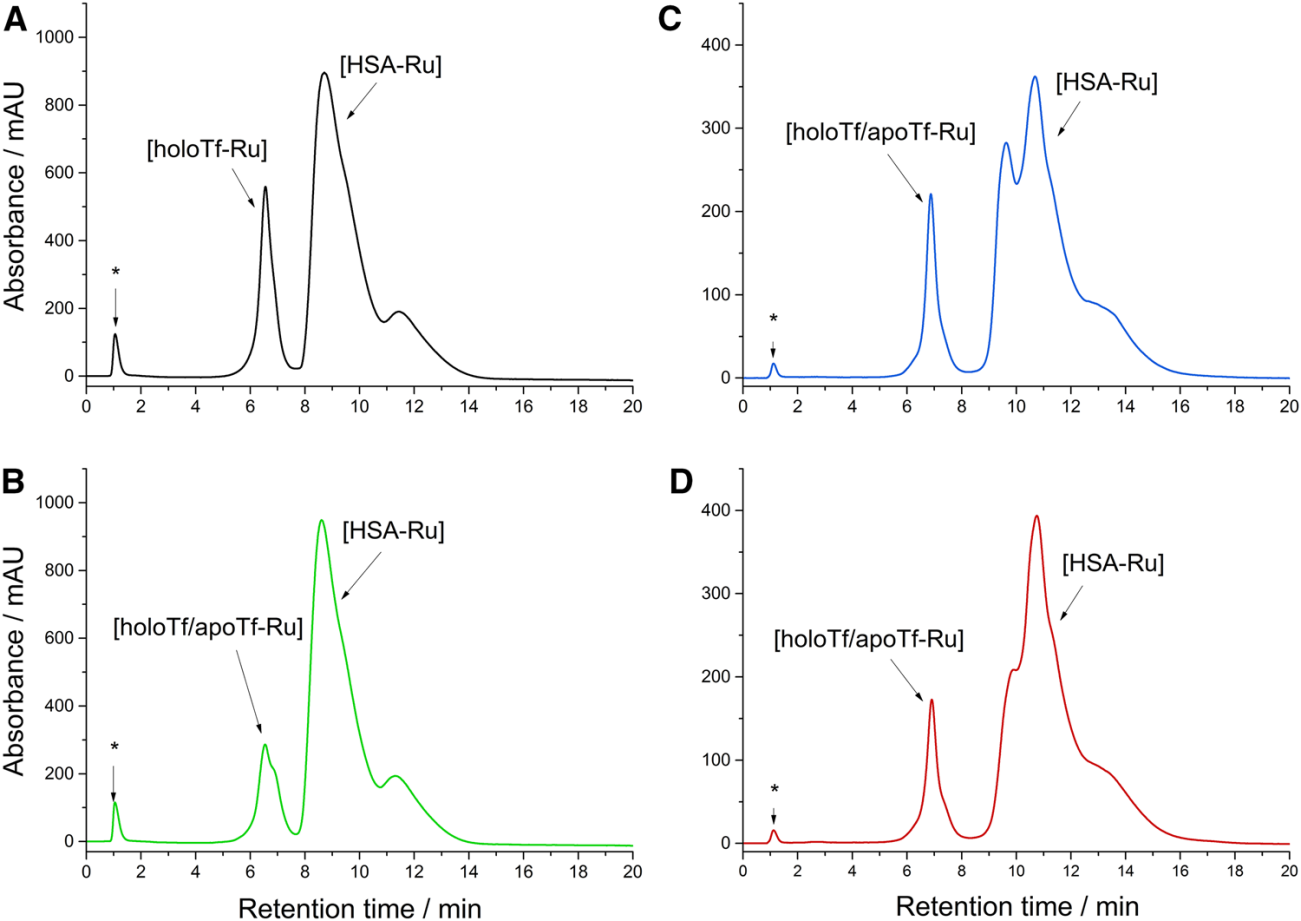
MonoQ chromatographic separation of serum models incubated with the 20-fold excess of NAMI-A over Tf for 24 h at 37 °C. **a** Model 0*, **b** Model 0, **c** Model 1 and **d** Model 2 (compositions are described in Fig. 5). *Asterisk* denotes the peak from solvent front comprising unbound Ru species. The elution conditions were described at the experimental part

NAMI-A at 20-fold excess over Tf (0.7 mM) was incubated with all these serum models for 24 h at 37 °C and then the reaction mixture was injected onto MonoQ column to separate unbound Ru species from those bound to Tf and HSA. The obtained chromatographs are presented in Fig. 6.

As can be seen in Table 2 Ru distribution varies between applied models. In Model 0* and Model 0, holo-Tf showed very similar binding properties pointing out that introducing apo form has a marginal impact on ruthenation of Tf. The presence of 18-fold excess of HSA over Tf has a distinct influence on holo-Tf–Ru adduct formation, lowering it by factor ca. two. Taking into account the fact that one molecule of HSA can bind ca. one and half Ru equivalent (data taken from [26]) all added Ru complex could be consumed by HSA. However, the obtained data proved that holo-Tf can readily compete in binding of Ru species. This very well correlates with data obtained from the intermolecular competitive binding studies showing that both proteins are ruthenated under deficiency of Ru complex (see Table 1). Calculation of the whole Ru distribution showed that in these two models, ca. 40–50 % of Ru species remained in the unbound form, despite equimolar concentration of NAMI-A and proteins. The addition of low-molecular-weight components of human serum included in Model 1 gave rise to evident increase in fraction of Ru equivalents in both proteins, while the fraction of free Ru species was negligible. The enhancement of Ru species binding to serum proteins can arise from transformation of NAMI-A into more active Ru species or/and modulation of protein surface making the binding sites more accessible. For instance, the reducing agent like cysteine could change the oxidation state of Ru or serve as a S-donor ligand. Moreover, N-donor ligands like His, or O-donors: Glu, oxalate, citrate and lactate can from other Ru complexes with higher binding affinity to serum proteins. Furthermore, in Model 2, where additionally Ca^2+^, Zn^2+^ and Mg^2+^ were included, Tf-bound fraction of Ru was even higher than in serum-like conditions without the addition of metal ions. The Ru fraction bound to HSA stayed on the similar level, while the fraction of free Ru species was minor. The introducing of metal ions did not decrease Ru binding, but, on the contrary, Tf-bound form was even more abundant. One can assume that they do not compete with Ru species in binding to Tf, what is more, they can influence the accessibility of Ru-binding sites by non-specific interaction with Tf. The crystal structure of Tf with Ca^2+^, Mg^2+^ and Zn^2+^ ligands is unknown and Tf does not have a primary role in the distribution of magnesium, copper, or zinc to tissues [46]. These interesting results need further clarification; however, it is beyond the scope of this work. In summary, the presence of only very small fraction of unbound Ru species in Model 1 and 2 indicates the high affinity of NAMI-A towards both serum proteins (HSA, Tf) and despite the presence of much higher amount of HSA over Tf still the formation of Tf–Ru adducts is feasible under physiological concentration of these proteins.

**Table 2:** Ru fraction [mol/mol (protein)] in ruthenated Tf or HSA produced after incubation of NAMI-A, Tf and HSA at 20:1:18 molar ratio for 24 h at 37 °C in various models of serum (the composition is depicted in Fig. 5)

Model	Tf saturation (%)	Ru/Tf (mol/mol)	Ru/HSA (mol/mol)
0*	100	0.50 ± 0.08	0.50 ± 0.06
0	30	0.52 ± 0.07	0.51 ± 0.05
1	30	2.0 ± 0.2	1.2 ± 0.1
2	30	2.9 ± 0.5	1.1 ± 0.1

### NAMI-A interaction with human serum

**Fig. 7:**
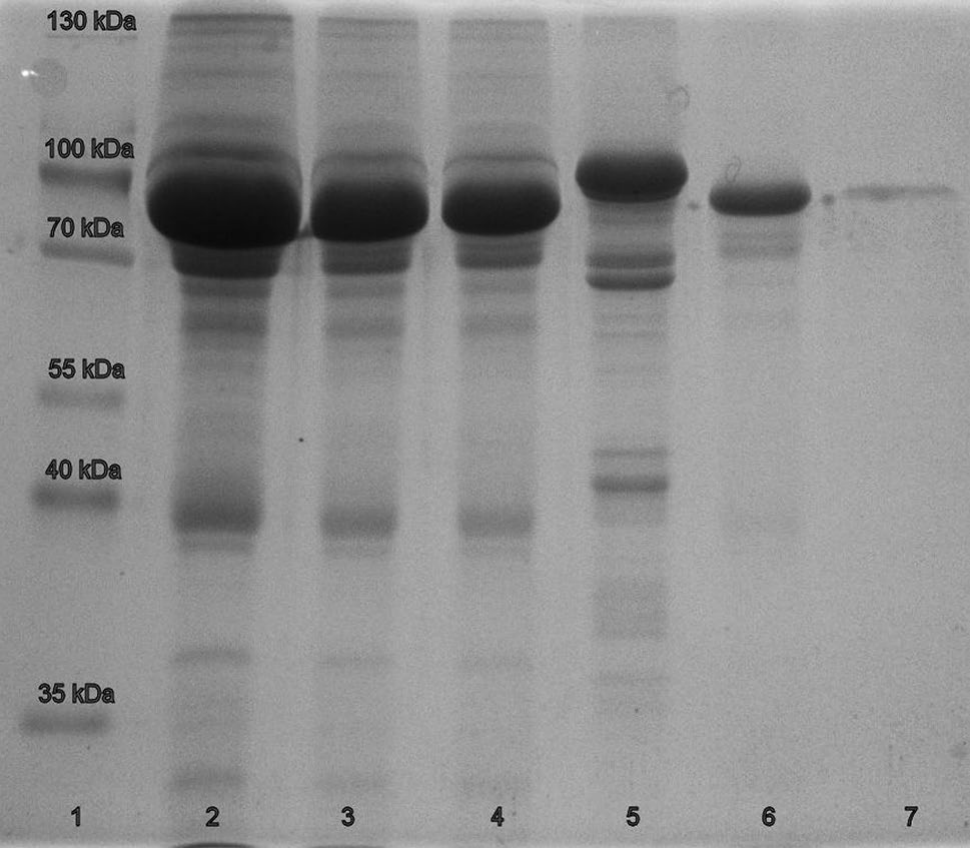
SDS-PAGE electrophoresis. *Line 1* protein marker; *lines 2*, *3* and *4* human serum diluted 10, 20 and 30 times, respectively; *line 5* human serum transferrin (Sigma-Aldrich) 40 μg; *line 6* unbound fraction of serum eluted from Capture Select resins; *line 7* elution fraction from Capture Select resins

The relevant physiological conditions have been achieved by application of human serum. Incubation of NAMI-A with human serum (20-fold excess of Ru complex over Tf) was performed over 24 h at 37 °C, and then Tf and ruthenated Tf were isolated using affinity chromatography resins (see Fig. S1). The efficiency of affinity column was checked by SDS-PAGE analysis (Fig. 7). SDS-PAGE gel showed that after elution only Tf was present. In separated Tf fraction, Ru and Tf concentrations were determined using ICP-MS and ELISA techniques, respectively. The analysis showed that 0.37 ± 0.03 mol of Ru was bound per mole of Tf. The presence of low-molecular-mass components of serum Models 1 and 2 increased Ru binding; however, in biological environment of blood, other proteins like immunoglobulins and fibrinogen can compete with Tf in binding of Ru species lowering the ruthenated Tf fraction. To verify if the macromolecules present in the serum can become a potential targets for NAMI-A, the overall amount of Ru species bound to proteins was assessed using ultrafiltration as a separation method. The mixture of NAMI-A and human serum incubated under the same conditions as previously, was centrifuged through the 3 K MWCO cutoff filter and both ultrafiltrate and protein fractions were collected for ICP-MS analysis. The fraction of Ru bound to proteins was found to be of 98 % (2 µg of Ru/1 mg of protein) proving that most of the Ru species exist in protein-bound form, and that the decrease of Ru in Tf fraction arises from binding to other proteins. These results are consistent with the data obtained from human clinical trials [34] as well as studies on combination therapy involving cisplatin and NAMI-A [47].

## Conclusions

To summarize, it was demonstrated that NAMI-A can bind to holo-Tf as readily as to apo-Tf without effecting iron content in this protein. In addition, the competition experiments showed that the affinity towards two serum proteins, namely HSA and transferrin is very similar. Although the concentration of HSA in human plasma is much higher than Tf, the ruthenated Tf was still detected under physiological-like conditions. Studies on serum models clearly showed the strong influence of low-molecular-mass components of serum on Ru complex binding to Tf. The additional proteins present in human serum effectively competed in Ru complex binding lowering the fraction of ruthenated Tf. In general, it was proved that protein-bound Ru forms are major metabolites of NAMI-A after administration to human serum. The presented results indicate that careful attention should be given to performing experiments under conditions better simulated real physiological environment. Presuming that ruthenated holo-Tf might be one of NAMI-A complex metabolites, further investigations on interaction of this adduct with TfR and its taking up into the cells via endocytosis seem to be rational research issue.

Electronic Supplementary Information (ESI): Affinity chromatography isolation of Tf from human serum, MonoQ chromatographic separation of holo-Tf incubated with NAMI-A, or HSA and Tf with NAMI-A at 1:1:1 molar ratio.

## Electronic supplementary material

Below is the link to the electronic supplementary material.

Supplementary material 1 (PDF 739 kb)
