# Supplementary material for: Impact of low- and high-molecular-mass components of human serum on NAMI-A binding to transferrin
Source: J Biol Inorg Chem. 2015 Mar 20;20(4):695–703. doi: 10.1007/s00775-015-1255-5 (PMC4436686; doi:10.1007/s00775-015-1255-5)
Supplement: Supplementary file 1 — Supplementary material 1 (PDF 739 kb) [file 775_2015_1255_MOESM1_ESM.pdf]

Electronic Supplementary Material

Bio-activation of 4-Alkyl Analogues of 1,4-Dihydropyridine Mediated by Cytochrome P450 Enzymes

Xiao-Xi Li,[1] Xiaoqian Zhang,[2] Qing-Chuan Zheng,[1] Yong Wang[3,✉]

---

[1] Xiao-Xi Li, Qing-Chuan Zheng

International Joint Research Laboratory of Nano-Micro Architecture Chemistry, State Key Laboratory of Theoretical and Computational Chemistry, Institute of Theoretical Chemistry, Jilin University,

Changchun 130023, People's Republic of China

[2] Xiaoqian Zhang

College of Physics and Electronic Engineer, Henan Normal University,

Xinxiang 453007, People's Republic of China

[3] Yong Wang (✉)

State Key Laboratory for Oxo Synthesis and Selective Oxidation, Lanzhou Institute of Chemical Physics, Chinese Academy of Sciences,

Lanzhou 730000, China

Email: wangyong@licp.cas.cn

Part I Proposed metabolic mechanisms

Scheme S1 All possible metabolic pathways for the oxidation of 4-alkyl substituted 3,5-bis(ethoxycarbonyl)-2,6-dimethyl-1,4-dihydropyridine by Cpd I.

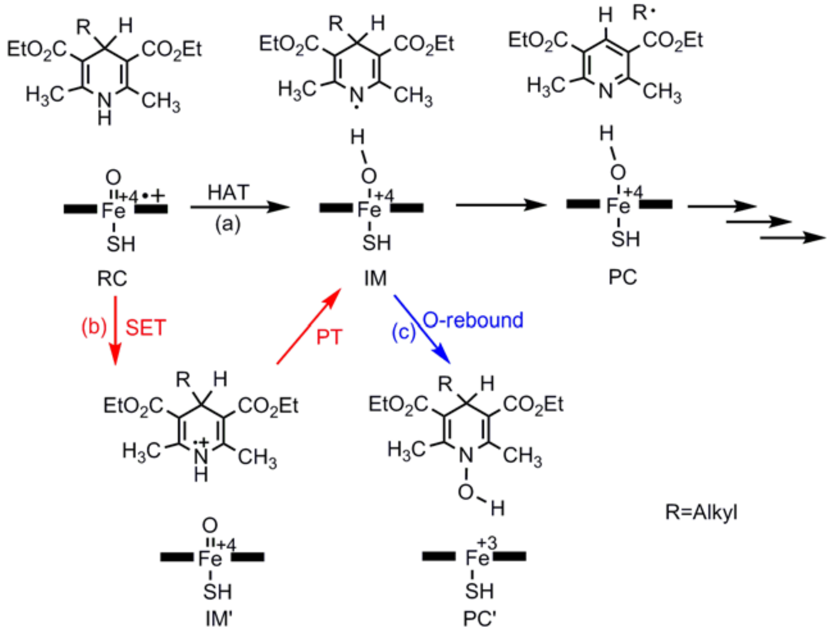

Part II The metabolism of DDEP by Cpd I

Table S1 Mulliken group spin densities and NBO charges for key moieties during the deethylation of DDEP by Cpd I of P450. All data were computed at the UB3LYP/B1 level. The $total_{ox}$ represents the NBO charges on the Cpd I moiety, while the $total_{sub}$ represents the NBO charges on the substrate group.

	Mulliken spin densities							NBO charges								
	Fe	O	Por	SH	H	Et	others	Fe	O	Por	SH	H	Et	others	$total_{ox}$	$total_{sub}$
^{4}**RC**	1.17	0.85	0.47	0.50	0.00	0.00	0.02	0.31	-0.29	0.07	-0.07	0.45	0.02	-0.48	0.02	-0.02
^{2}**RC**	1.29	0.81	-0.55	-0.56	0.00	0.00	0.00	0.32	-0.30	0.07	-0.06	0.45	0.01	-0.49	0.03	-0.03
^{4}**TS**1	1.50	0.55	0.05	0.13	-0.01	0.07	0.71	0.35	-0.54	-0.19	-0.12	0.48	0.07	-0.04	-0.51	0.51
^{2}**TS**1	1.62	0.50	-0.30	-0.09	0.00	-0.07	-0.66	0.37	-0.56	-0.15	-0.16	0.48	0.07	-0.06	-0.50	0.50
^{4}**IM**	1.74	0.29	-0.12	0.09	0.00	0.09	0.91	0.39	-0.65	-0.22	-0.05	0.50	0.08	-0.04	-0.54	0.54
^{2}**IM**	1.74	0.29	-0.12	0.09	0.01	-0.09	-0.91	0.39	-0.65	-0.22	-0.05	0.50	0.08	-0.04	-0.54	0.54
^{4}**TS**2	1.74	0.30	-0.12	0.08	0.00	0.68	0.32	0.39	-0.65	-0.23	-0.06	0.50	0.11	-0.07	-0.54	0.54
^{2}**TS**2	1.74	0.30	-0.12	0.08	0.00	-0.68	-0.31	0.39	-0.65	-0.23	-0.06	0.50	0.11	-0.07	-0.54	0.54
^{4}**PC**	1.75	0.29	-0.12	0.07	0.00	0.99	0.01	0.40	-0.66	-0.22	-0.05	0.50	0.00	0.03	-0.54	0.54
^{2}**PC**	1.75	0.29	-0.12	0.08	0.00	-0.99	0.00	0.39	-0.66	-0.22	-0.05	0.50	-0.01	0.04	-0.53	0.53

Table S2 The deethylation of DDEP mediated by Cpd I. SCF energies at various computational levels. Absolute SCF energies are in Hartree and relative SCF energies are in kcal/mol.

	OPT/B1		OPT/B1+ZPE		SPE/B2		SPE/B2+ZPE		SOLV		SOLV+ZPE	
^{4}**RC**	-2526.917185	0.0	-2526.260945	0.0	-2527.544813	0.0	-2526.888573	0.0	-2527.564330	0.0	-2526.908090	0.0
^{2}**RC**	-2526.917284	-0.1	-2526.261064	-0.1	-2527.544994	-0.1	-2526.888774	-0.1	-2527.564440	-0.1	-2526.908220	-0.1
^{4}**TS**1	-2526.900862	10.2	-2526.251503	5.9	-2527.532930	7.5	-2526.883571	3.1	-2527.553134	7.0	-2526.903775	2.7
^{2}**TS**1	-2526.900595	10.4	-2526.250903	6.3	-2527.532658	7.6	-2526.882966	3.5	-2527.553092	7.1	-2526.903400	2.9
^{4}**IM**	-2526.914865	1.5	-2526.261221	-0.2	-2527.548832	-2.5	-2526.895188	-4.2	-2527.567235	-1.8	-2526.913591	-3.5
^{2}**IM**	-2526.914844	1.5	-2526.261200	-0.2	-2527.548822	-2.5	-2526.895178	-4.2	-2527.567231	-1.8	-2526.913587	-3.4
^{4}**TS**2	-2526.891248	16.3	-2526.240670	12.7	-2527.527073	11.1	-2526.876495	7.6	-2527.546036	11.5	-2526.895458	7.9
^{2}**TS**2	-2526.891245	16.3	-2526.240671	12.7	-2527.527074	11.1	-2526.876500	7.6	-2527.546041	11.5	-2526.895467	7.9
^{4}**PC**	-2526.904260	8.1	-2526.255944	3.1	-2527.542493	1.5	-2526.894177	-3.5	-2527.559638	2.9	-2526.911322	-2.0
^{2}**PC**	-2526.903076	8.9	-2526.254767	3.9	-2527.540960	2.4	-2526.892651	-2. 6	-2527.559308	3.2	-2526.910999	-1.8

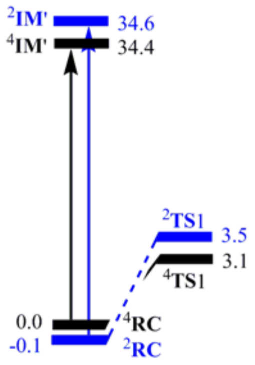

Fig. S1 Energy profiles (in kcal/mol) for the initial H-abstraction process during the N-deethylation of DDEP by Cpd I on the quartet and doublet spin states in vacuum. All energy data are calculated at the UB3LYP/B2//B1 level, including zero point vibrational energy (ZPE) correction.

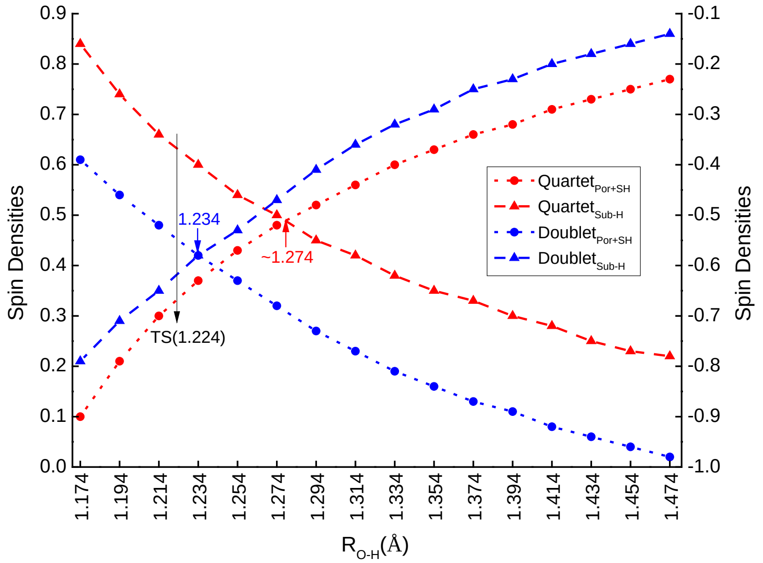

Fig. S2 Changes in the spin densities (ρ) on Porphine+thiolate (Por+SH, dot line) and Substrate-hydrogen (Sub-H, dash line) moieties as a function of the O-H distance (red for quartet spin state, blue for doublet spin state). The spin density on Fe-O moiety keeps *ca*. 2.0.

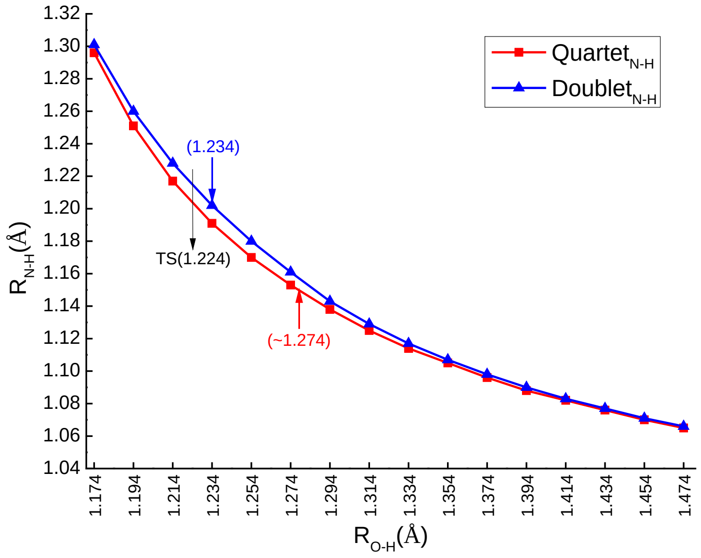

Fig. S3 Change of the N-H distance as a function of the O-H distance (red for quartet spin state, blue for doublet spin state). All distances are in Å.

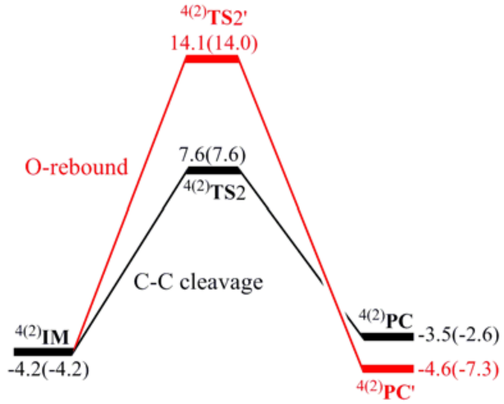

Fig. S4 Energy profiles (in kcal/mol) for the homolytic scission of C-C bond (in black) and O-rebound processes (in red) during the deethylation reaction of DDEP calculated with UB3LYP/B2//B1 on the quartet (doublet) state in vaccum. All energy data include zero point vibrational energy (ZPE) correction.

Part III The effects of substituent at 4-carbon on the reaction

III-1. The metabolism of DDC by Cpd I

Table S3 Mulliken group spin densities and NBO charges for key moieties during the demethylation of DDC by Cpd I of P450. All data were computed at the UB3LYP/B1 level. The $total_{ox}$ represents the NBO charges on the Cpd I moiety, while the $total_{sub}$ represents the NBO charges on the substrate group.

	Mulliken spin densities							NBO charges								
	Fe	O	Por	SH	H	Et	others	Fe	O	Por	SH	H	Et	others	$total_{ox}$	$total_{sub}$
^{4}**RC**	1.17	0.85	0.47	0.50	0.00	0.00	0.01	0.31	-0.29	0.08	-0.06	0.45	0.02	-0.49	0.03	-0.03
^{2}**RC**	1.30	0.80	-0.55	-0.55	0.00	0.00	0.01	0.32	-0.30	0.07	-0.06	0.45	0.02	-0.49	0.03	-0.03
^{4}**TS**1	1.50	0.55	0.05	0.13	-0.01	0.06	0.72	0.34	-0.54	-0.18	-0.13	0.48	0.07	-0.04	-0.50	0.50
^{2}**TS**1	1.56	0.52	-0.33	-0.02	0.00	-0.06	-0.67	0.35	-0.55	-0.14	-0.17	0.48	0.07	-0.05	-0.50	0.50
^{4}**IM**	1.74	0.29	-0.12	0.08	0.00	0.08	0.93	0.39	-0.65	-0.22	-0.05	0.50	0.07	-0.04	-0.54	0.54
^{2}**IM**	1.74	0.29	-0.12	0.08	0.01	-0.08	-0.92	0.40	-0.65	-0.22	-0.05	0.50	0.07	-0.04	-0.54	0.54
^{4}**TS**2	1.74	0.30	-0.12	0.08	0.00	0.73	0.28	0.39	-0.65	-0.23	-0.05	0.50	0.07	-0.04	-0.54	0.54
^{2}**TS**2	1.74	0.30	-0.12	0.08	0.00	-0.73	-0.27	0.39	-0.65	-0.23	-0.05	0.50	0.07	-0.04	-0.54	0.54
^{4}**PC**	1.75	0.29	-0.12	0.08	0.00	1.00	0.01	0.40	-0.66	-0.22	-0.05	0.50	-0.01	0.04	-0.53	0.53
^{2}**PC**	1.75	0.29	-0.12	0.08	0.00	-1.00	0.00	0.40	-0.66	-0.22	-0.05	0.50	-0.01	0.04	-0.53	0.53

Table S4 The demethylation of DDC by Cpd I of P450. SCF energies at various computational levels. Absolute SCF energies are in Hartree and relative SCF energies are in kcal/mol.

	OPT/B1		OPT/B1+ZPE		SPE/B2		SPE/B2+ZPE		SOLV		SOLV+ZPE	
^{4}**RC**	-2487.606652	0.00	-2486.978937	0.00	-2488.222238	0.00	-2487.594523	0.00	-2488.241420	0.0	-2487.613705	0.0
^{2}**RC**	-2487.606747	-0.06	-2486.979027	-0.06	-2488.222341	-0.06	-2487.594621	-0.06	-2488.241519	-0.1	-2487.613799	-0.1
^{4}**TS**1	-2487.590850	9.92	-2486.969780	5.75	-2488.211894	6.49	-2487.590824	2.32	-2488.231762	6.1	-2487.610692	1.9
^{2}**TS**1	-2487.590322	10.25	-2486.969159	6.14	-2488.211237	6.90	-2487.590074	2.79	-2488.231773	6.1	-2487.610610	1.9
^{4}**IM**	-2487.604891	1.10	-2486.979458	-0.33	-2488.227769	-3.47	-2487.602336	-4.90	-2488.245988	-2.9	-2487.620555	-4.3
^{2}**IM**	-2487.604874	1.12	-2486.979446	-0.32	-2488.227764	-3.47	-2487.602336	-4.90	-2488.245991	-2.9	-2487.620563	-4.3
^{4}**TS**2	-2487.573781	20.63	-2486.952260	16.74	-2488.199074	14.54	-2487.577553	10.65	-2488.217499	15.0	-2487.595978	11.1
^{2}**TS**2	-2487.573779	20.63	-2486.952257	16.74	-2488.199077	14.53	-2487.577555	10.65	-2488.217504	15.0	-2487.595982	11.1
^{4}**PC**	-2487.584245	14.06	-2486.965432	8.47	-2488.211997	6.43	-2487.593184	0.84	-2488.230142	7.1	-2487.611329	1.5
^{2}**PC**	-2487.584245	14.06	-2486.965427	8.48	-2488.211996	6.43	-2487.593178	0.84	-2488.230137	7.1	-2487.611319	1.5

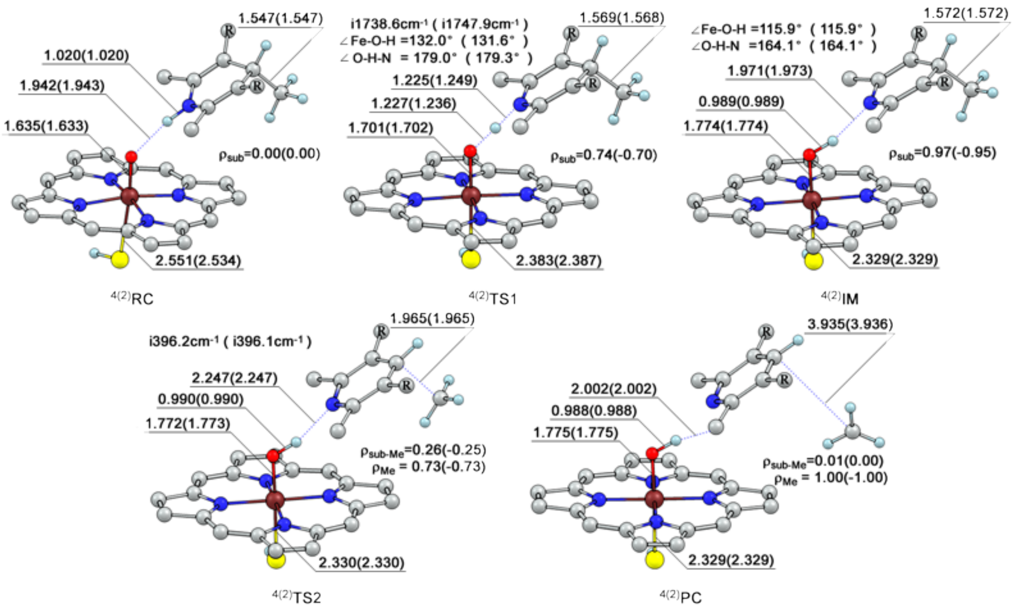

Fig. S5 Conformations of various reaction species (lengths in Å) optimized at the UB3LYP/B1 level. For $^{4(2)}$**TS**s, imaginary frequencies (in cm^{-1}) were included. Some important angles (in ˚) and part of group spin densities (ρ) were included. Each data set gives HS and LS values. Unimportant hydrogen atoms are omitted for clarity. R is equal to ethoxycarbonyl group.

III-2. The metabolism of DDIP by Cpd I

Table S5 Mulliken group spin densities and NBO charges for key moieties during the deisopropylation of DDIP by Cpd I of P450. All data were computed at the UB3LYP/B1 level. The $total_{ox}$ represents the NBO charges on the Cpd I moiety, while the $total_{sub}$ represents the NBO charges on the substrate group.

	Mulliken spin densities							NBO charges								
	Fe	O	Por	SH	H	Et	others	Fe	O	Por	SH	H	Et	others	$total_{ox}$	$total_{sub}$
^{4}**RC**	1.17	0.85	0.47	0.50	0.00	0.00	0.01	0.31	-0.29	0.08	-0.06	0.45	0.01	-0.49	0.03	-0.03
^{2}**RC**	1.30	0.80	-0.55	-0.56	0.00	0.00	0.01	0.32	-0.30	0.07	-0.06	0.45	0.01	-0.49	0.03	-0.03
^{4}**TS**1	1.53	0.53	0.07	0.10	-0.01	0.08	0.70	0.36	-0.55	-0.17	-0.14	0.48	0.07	-0.05	-0.50	0.50
^{2}**TS**1	1.61	0.51	-0.33	-0.09	0.00	-0.08	-0.62	0.37	-0.55	-0.12	-0.16	0.48	0.07	-0.08	-0.47	0.47
^{4}**IM**	1.74	0.29	-0.12	0.08	0.00	0.11	0.90	0.39	-0.65	-0.22	-0.05	0.50	0.08	-0.05	-0.54	0.54
^{2}**IM**	1.74	0.29	-0.12	0.08	0.01	-0.11	-0.89	0.39	-0.65	-0.22	-0.05	0.50	0.08	-0.05	-0.54	0.54
^{4}**TS**2	1.74	0.30	-0.12	0.08	0.00	0.62	0.39	0.39	-0.65	-0.23	-0.06	0.50	0.14	-0.10	-0.04	0.04
^{2}**TS**2	1.74	0.30	-0.12	0.08	0.00	-0.62	-0.38	0.39	-0.65	-0.23	-0.06	0.50	0.14	-0.10	-0.04	0.04
^{4}**PC**	1.75	0.29	-0.12	0.08	0.00	1.00	0.01	0.40	-0.66	-0.22	-0.05	0.50	-0.01	0.04	-0.03	0.03
^{2}**PC**	1.75	0.29	-0.12	0.08	0.00	-1.00	0.00	0.40	-0.66	-0.22	-0.05	0.50	-0.01	0.04	-0.03	0.03

Table S6 The deisopropylation of DDIP by Cpd I of P450. SCF energies at various computational levels. Absolute SCF energies are in Hartree and relative SCF energies are in kcal/mol.

	OPT/B1		OPT/B1+ZPE		SPE/B2		SPE/B2+ZPE		SOLV		SOLV+ZPE	
^{4}**RC**	-2566.228820	0.00	-2565.544219	0.00	-2566.866419	0.00	-2566.181818	0.00	-2566.885471	0.0	-2566.200870	0.0
^{2}**RC**	-2566.228932	-0.07	-2565.544378	-0.10	-2566.866567	-0.09	-2566.182013	-0.12	-2566.885595	-0.1	-2566.201041	-0.1
^{4}**TS**1	-2566.210253	11.65	-2565.532316	7.47	-2566.853344	8.20	-2566.175407	4.02	-2566.873557	7.5	-2566.195620	3.3
^{2}**TS**1	-2566.210093	11.75	-2565.532305	7.48	-2566.853054	8.39	-2566.175266	4.11	-2566.873035	7.8	-2566.195247	3.5
^{4}**IM**	-2566.223961	3.05	-2565.541683	1.59	-2566.869036	-1.64	-2566.186758	-3.10	-2566.887392	-1.2	-2566.205114	-2.7
^{2}**IM**	-2566.223935	3.07	-2565.541656	1.61	-2566.869028	-1.64	-2566.186749	-3.09	-2566.887389	-1.2	-2566.205110	-2.7
^{4}**TS**2	-2566.207459	13.40	-2565.527782	10.31	-2566.853989	7.80	-2566.174312	4.71	-2566.873235	7.7	-2566.193558	4.6
^{2}**TS**2	-2566.207455	13.41	-2565.527776	10.32	-2566.853991	7.80	-2566.174312	4.71	-2566.873243	7.7	-2566.193564	4.6
^{4}**PC**	-2566.223240	3.50	-2565.546018	-1.13	-2566.871891	-3.43	-2566.194669	-8.06	-2566.889930	-2.8	-2566.212708	-7.4
^{2}**PC**	-2566.223239	3.50	-2565.546018	-1.13	-2566.871890	-3.43	-2566.194669	-8.06	-2566.889929	-2.8	-2566.212708	-7.4

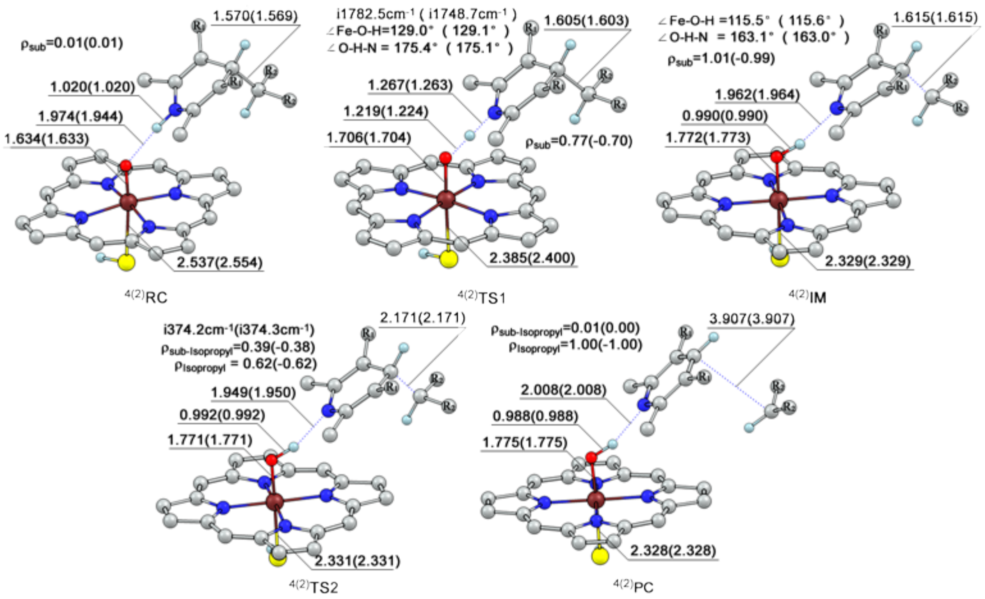

Fig. S6 Conformations of various reaction species (lengths in Å) optimized at the UB3LYP/B1 level. For $^{4(2)}$**TS**s, imaginary frequencies (in cm^{-1}) were included. Some important angles (in ˚) and part of group spin densities (ρ) were included. Each data set gives the quartet and doublet spin state values. Unimportant hydrogen atoms are omitted for clarity. R_1 is equal to ethoxycarbonyl group and R_2 is equal to methyl group.

Table S7 N-H and C-C bond dissociation energies (in kcal/mol) of three substrates calculated at the UB3LYP/B2 level, with ZPE correction incorporated.

Substrates	BDE_{N-H}	BDE_{C-C}
DDC	81.7	54.2
DDEP	82.3	50.2
DDIP	83.7	45.6

Part IV. Cartesian Coordinates of the Key Reaction Species.

A. Demethylation of DDC

^{4}RC

Fe	6.833490000	-13.802129000	21.905324000
N	5.547276000	-12.945931000	23.203470000
C	5.757867000	-12.721727000	24.540748000
C	4.560650000	-12.206327000	25.157890000
C	3.619001000	-12.128722000	24.178022000
C	4.245924000	-12.593843000	22.965164000
H	4.469817000	-11.946048000	26.204515000
H	2.593658000	-11.789876000	24.251513000
C	3.614045000	-12.652146000	21.726163000
H	2.575682000	-12.338033000	21.682489000
N	5.498925000	-13.518914000	20.418354000
C	4.200751000	-13.072918000	20.544314000
C	3.528951000	-13.127056000	19.269496000
C	4.421617000	-13.628195000	18.374311000
C	5.644033000	-13.878784000	19.096788000
H	2.503976000	-12.822249000	19.101724000
H	4.282788000	-13.819250000	17.318038000
C	6.791242000	-14.416857000	18.537383000
H	6.761519000	-14.666272000	17.481286000
N	8.200229000	-14.390862000	20.542984000
C	7.985708000	-14.639016000	19.211442000
C	9.185858000	-15.149716000	18.595166000
C	10.133099000	-15.206525000	19.569656000
C	9.508537000	-14.738690000	20.782785000
H	9.274527000	-15.419302000	17.550750000
H	11.161548000	-15.535009000	19.493944000
C	10.140722000	-14.670065000	22.014644000
H	11.179523000	-14.982121000	22.059392000
N	8.260811000	-13.810112000	23.329001000
C	9.551450000	-14.243978000	23.201085000
C	10.233611000	-14.183423000	24.470949000
C	9.333923000	-13.703120000	25.372316000
C	8.108423000	-13.470995000	24.647585000
H	11.263960000	-14.472391000	24.632401000
H	9.470440000	-13.516108000	26.429479000
C	6.948116000	-12.959020000	25.215282000
H	6.972023000	-12.732216000	26.276711000
O	6.330273000	-15.317734000	22.255834000
S	7.528620000	-11.502551000	21.098243000
H	6.397739000	-10.838923000	21.429267000
C	6.954387000	-17.630343000	25.229706000
C	7.580309000	-18.516603000	26.060010000
C	8.876730000	-19.185683000	25.614796000
C	8.907490000	-19.325395000	24.096144000
C	8.241245000	-18.421957000	23.317288000
N	7.401596000	-17.506596000	23.926579000
C	8.272966000	-18.329420000	21.812537000
C	5.756879000	-16.775368000	25.557794000
C	9.709122000	-20.381174000	23.470157000
O	9.867373000	-20.575579000	22.271419000
O	10.305308000	-21.184125000	24.402168000
C	11.095644000	-22.264489000	23.878885000
C	11.653840000	-23.035827000	25.062159000
C	7.046364000	-18.765792000	27.402435000
O	6.054275000	-18.256414000	27.908755000
O	7.799326000	-19.679794000	28.085163000
C	7.332067000	-20.011010000	29.403413000
C	8.276580000	-21.054889000	29.973432000
C	10.109322000	-18.403860000	26.128730000
H	8.921558000	-20.182712000	26.055583000
H	6.954490000	-16.806266000	23.335424000
H	9.298254000	-18.312327000	21.438343000
H	7.803638000	-19.211082000	21.364308000
H	7.741465000	-17.435378000	21.473503000
H	5.573914000	-16.051403000	24.758096000
H	4.863400000	-17.396524000	25.680717000
H	5.893125000	-16.253156000	26.506254000
H	10.466582000	-22.894843000	23.240847000
H	11.891151000	-21.855598000	23.246149000
H	12.265475000	-23.873657000	24.708757000
H	12.279714000	-22.391594000	25.688615000
H	10.845748000	-23.435947000	25.683344000
H	6.303855000	-20.383143000	29.338098000
H	7.309170000	-19.103293000	30.016606000
H	7.956693000	-21.338604000	30.982473000
H	9.299037000	-20.667161000	30.031750000
H	8.286908000	-21.954257000	29.348885000
H	11.035050000	-18.906191000	25.825264000
H	10.093224000	-18.341520000	27.222935000
H	10.117832000	-17.384275000	25.724631000

^{2}RC

Fe	6.855250000	-13.817304000	21.905734000
N	5.596795000	-12.970717000	23.235011000
C	5.830079000	-12.768269000	24.571920000
C	4.646527000	-12.255292000	25.216372000
C	3.689673000	-12.156956000	24.253221000
C	4.293203000	-12.608039000	23.023380000
H	4.574407000	-12.010310000	26.268128000
H	2.667811000	-11.813111000	24.348657000
C	3.637483000	-12.648383000	21.796218000
H	2.599498000	-12.330565000	21.777241000
N	5.493364000	-13.503355000	20.441648000

C	4.197962000	-13.060071000	20.598500000
C	3.498701000	-13.106486000	19.338146000
C	4.371734000	-13.603120000	18.421269000
C	5.609377000	-13.857977000	19.116084000
H	2.470396000	-12.800767000	19.194112000
H	4.209985000	-13.788530000	17.367240000
C	6.744529000	-14.393142000	18.529738000
H	6.692963000	-14.635075000	17.472729000
N	8.194886000	-14.387643000	20.506862000
C	7.951433000	-14.624198000	19.177942000
C	9.137129000	-15.133235000	18.532718000
C	10.104237000	-15.201243000	19.486755000
C	9.505956000	-14.742305000	20.716387000
H	9.203323000	-15.394039000	17.484399000
H	11.129779000	-15.532363000	19.387076000
C	10.162064000	-14.686990000	21.936474000
H	11.201309000	-14.999948000	21.958228000
N	8.305367000	-13.846296000	23.297662000
C	9.595314000	-14.274759000	23.138247000
C	10.302322000	-14.227973000	24.394611000
C	9.419523000	-13.762703000	25.320315000
C	8.179200000	-13.524847000	24.624008000
H	11.336600000	-14.515503000	24.531517000
H	9.576965000	-13.588567000	26.376757000
C	7.030629000	-13.021319000	25.221791000
H	7.074748000	-12.811451000	26.286027000
O	6.333631000	-15.334669000	22.209250000
S	7.530256000	-11.496260000	21.090239000
H	6.408038000	-10.837744000	21.458719000
C	6.906554000	-17.651880000	25.191186000
C	7.531081000	-18.524186000	26.037023000
C	8.848950000	-19.170668000	25.621510000
C	8.915687000	-19.311612000	24.103915000
C	8.249700000	-18.422255000	23.308871000
N	7.379007000	-17.522548000	23.897510000
C	8.311009000	-18.332290000	21.804907000
C	5.685235000	-16.820091000	25.490287000
C	9.750454000	-20.353262000	23.498028000
O	9.935092000	-20.550271000	22.303464000
O	10.346111000	-21.139092000	24.445028000
C	11.168001000	-22.205224000	23.941756000
C	11.722213000	-22.957780000	25.138934000
C	6.972931000	-18.780759000	27.368067000
O	5.960202000	-18.290125000	27.851598000
O	7.728958000	-19.678054000	28.069610000
C	7.240154000	-20.015139000	29.378413000
C	8.190686000	-21.041353000	29.970177000
C	10.056427000	-18.367821000	26.161680000
H	8.900773000	-20.166561000	26.064244000
H	6.935035000	-16.828942000	23.296296000
H	9.343089000	-18.316249000	21.450532000
H	7.849560000	-19.214537000	21.349190000
H	7.785484000	-17.439178000	21.454201000
H	5.510781000	-16.096049000	24.688619000
H	4.800188000	-17.458082000	25.585755000
H	5.785618000	-16.299925000	26.444190000
H	10.563557000	-22.852491000	23.296883000
H	11.965383000	-21.783087000	23.320164000
H	12.358135000	-23.783860000	24.801051000
H	12.322858000	-22.296559000	25.772357000
H	10.912403000	-23.372173000	25.748418000
H	6.220169000	-20.405295000	29.292633000
H	7.188733000	-19.106946000	29.989215000
H	7.855369000	-21.328224000	30.973303000
H	9.204973000	-20.635993000	30.048071000
H	8.229059000	-21.941825000	29.348321000
H	10.996918000	-18.855664000	25.880796000
H	10.014446000	-18.303722000	27.255105000
H	10.058026000	-17.349107000	25.755418000

^{4}**TS**1

Fe	6.916638000	-14.012690000	21.947074000
N	5.719587000	-13.253454000	23.387380000
C	6.013634000	-13.117284000	24.719723000
C	4.865037000	-12.607655000	25.429714000
C	3.873127000	-12.450125000	24.510425000
C	4.414401000	-12.862330000	23.237687000
H	4.838156000	-12.406997000	26.493261000
H	2.862606000	-12.092640000	24.662300000
C	3.699720000	-12.866621000	22.046381000
H	2.669349000	-12.526713000	22.082512000
N	5.465657000	-13.720507000	20.577653000
C	4.196172000	-13.271722000	20.814904000
C	3.436517000	-13.257967000	19.584894000
C	4.270282000	-13.700513000	18.606147000
C	5.539259000	-13.983535000	19.238237000
H	2.403976000	-12.943141000	19.502698000
H	4.067477000	-13.825421000	17.550072000
C	6.667332000	-14.431766000	18.564344000
H	6.581682000	-14.586922000	17.493373000
N	8.195827000	-14.551185000	20.480895000
C	7.901172000	-14.686195000	19.149360000
C	9.067453000	-15.137403000	18.428874000
C	10.066878000	-15.272356000	19.343136000
C	9.511488000	-14.907643000	20.624508000
H	9.099115000	-15.319427000	17.362179000
H	11.089480000	-15.589952000	19.183778000
C	10.216109000	-14.942091000	21.821440000
H	11.259441000	-15.238720000	21.777220000

N	8.408749000	-14.215050000	23.313623000
C	9.698016000	-14.611847000	23.066213000
C	10.461063000	-14.603582000	24.291832000
C	9.619578000	-14.183218000	25.276886000
C	8.341489000	-13.931926000	24.653979000
H	11.507896000	-14.869727000	24.366437000
H	9.832006000	-14.032536000	26.327828000
C	7.229003000	-13.431130000	25.316791000
H	7.320101000	-13.255790000	26.384348000
O	6.367877000	-15.600434000	22.211151000
S	7.766191000	-11.823950000	21.538396000
H	7.161658000	-11.180446000	22.562079000
C	6.729883000	-17.525406000	24.925081000
C	7.368589000	-18.337744000	25.846308000
C	8.671715000	-19.003801000	25.489192000
C	8.881654000	-19.098942000	24.000405000
C	8.183298000	-18.262300000	23.146560000
N	7.203221000	-17.447817000	23.644052000
C	8.352023000	-18.200719000	21.649734000
C	5.471754000	-16.735092000	25.180641000
C	9.863124000	-20.069824000	23.470139000
O	10.183636000	-20.205206000	22.300570000
O	10.387587000	-20.842572000	24.457565000
C	11.343822000	-21.838166000	24.033560000
C	11.794444000	-22.594931000	25.269352000
C	6.804467000	-18.533929000	27.199102000
O	5.808314000	-17.992656000	27.650624000
O	7.529752000	-19.429817000	27.919091000
C	7.041629000	-19.706847000	29.249702000
C	7.969425000	-20.733755000	29.872280000
C	9.866059000	-18.233010000	26.152372000
H	8.690778000	-20.009507000	25.919429000
H	6.791194000	-16.509303000	22.919101000
H	9.388678000	-17.998320000	21.374454000
H	8.100780000	-19.167877000	21.202940000
H	7.702978000	-17.429117000	21.233111000
H	5.214431000	-16.147252000	24.298316000
H	4.645207000	-17.411737000	25.420233000
H	5.585289000	-16.076677000	26.043660000
H	10.865986000	-22.495391000	23.299875000
H	12.177053000	-21.336403000	23.530772000
H	12.519852000	-23.365833000	24.987358000
H	12.270380000	-21.922469000	25.990545000
H	10.946593000	-23.083309000	25.760585000
H	6.012011000	-20.071972000	29.178151000
H	7.019115000	-18.772331000	29.819780000
H	7.629740000	-20.973610000	30.885760000
H	8.993605000	-20.351639000	29.934353000
H	7.981009000	-21.658005000	29.285508000
H	10.804405000	-18.746059000	25.925750000
H	9.731204000	-18.211272000	27.237126000
H	9.915572000	-17.207177000	25.775190000

^{2}**TS**1

Fe	6.933150000	-14.017151000	21.955128000
N	5.735226000	-13.265456000	23.398368000
C	6.035188000	-13.124481000	24.728392000
C	4.887172000	-12.623048000	25.444811000
C	3.887767000	-12.477496000	24.531618000
C	4.425178000	-12.886505000	23.256351000
H	4.865266000	-12.420216000	26.508021000
H	2.874972000	-12.129057000	24.689132000
C	3.704748000	-12.894046000	22.068183000
H	2.671151000	-12.564727000	22.111186000
N	5.473495000	-13.716546000	20.587642000
C	4.200554000	-13.282048000	20.830867000
C	3.438584000	-13.261768000	19.601958000
C	4.274187000	-13.688491000	18.618085000
C	5.545928000	-13.969237000	19.246393000
H	2.403712000	-12.953585000	19.524170000
H	4.070903000	-13.804784000	17.561144000
C	6.674189000	-14.411940000	18.569478000
H	6.587417000	-14.560556000	17.497607000
N	8.208285000	-14.543841000	20.481457000
C	7.910089000	-14.668636000	19.149860000
C	9.074393000	-15.113487000	18.422258000
C	10.076767000	-15.254235000	19.332473000
C	9.523835000	-14.899723000	20.617743000
H	9.102993000	-15.287309000	17.354140000
H	11.099296000	-15.569260000	19.167817000
C	10.230665000	-14.939615000	21.813852000
H	11.275182000	-15.231558000	21.765914000
N	8.424442000	-14.227928000	23.313794000
C	9.713886000	-14.618851000	23.062038000
C	10.483005000	-14.606219000	24.283907000
C	9.645047000	-14.184991000	25.271262000
C	8.363922000	-13.938683000	24.652495000
H	11.530973000	-14.868994000	24.354060000
H	9.861542000	-14.029917000	26.320697000
C	7.255073000	-13.434053000	25.318780000
H	7.352377000	-13.254288000	26.385077000
O	6.373615000	-15.606698000	22.196765000
S	7.730844000	-11.811364000	21.513225000
H	6.977050000	-11.143520000	22.414859000
C	6.722319000	-17.529174000	24.918893000
C	7.359990000	-18.339225000	25.843064000
C	8.664255000	-19.005481000	25.489138000
C	8.876824000	-19.103173000	24.000568000
C	8.179368000	-18.268148000	23.144168000

N	7.197854000	-17.454422000	23.638993000
C	8.350313000	-18.207783000	21.647450000
C	5.462992000	-16.739118000	25.169397000
C	9.858620000	-20.074776000	23.473204000
O	10.178763000	-20.213942000	22.303858000
O	10.384021000	-20.844133000	24.462932000
C	11.340147000	-21.840857000	24.041584000
C	11.791696000	-22.593527000	25.279546000
C	6.793087000	-18.533327000	27.194635000
O	5.793397000	-17.994680000	27.641781000
O	7.519862000	-19.424168000	27.919524000
C	7.028980000	-19.699098000	29.249485000
C	7.958779000	-20.720747000	29.877725000
C	9.857016000	-18.232409000	26.152288000
H	8.683297000	-20.010315000	25.921395000
H	6.789101000	-16.522294000	22.915115000
H	9.388566000	-18.012191000	21.373263000
H	8.093551000	-19.173540000	21.200618000
H	7.706001000	-17.432462000	21.230194000
H	5.212836000	-16.146629000	24.288014000
H	4.634012000	-17.416603000	25.398381000
H	5.569895000	-16.085902000	26.037101000
H	10.862047000	-22.500604000	23.310323000
H	12.173007000	-21.340631000	23.536637000
H	12.516952000	-23.365327000	24.999615000
H	12.268075000	-21.918659000	25.998197000
H	10.944203000	-23.080308000	25.772982000
H	6.000904000	-20.068211000	29.176162000
H	7.001430000	-18.763029000	29.816803000
H	7.617196000	-20.958800000	30.890995000
H	8.981389000	-20.334714000	29.941432000
H	7.975360000	-21.646706000	29.293773000
H	10.795964000	-18.745483000	25.928216000
H	9.720413000	-18.208075000	27.236761000
H	9.906904000	-17.207421000	25.772842000

^{4}IM

Fe	6.863468000	-13.847308000	21.807765000
N	5.677936000	-13.141519000	23.279581000
C	5.977157000	-13.070721000	24.617539000
C	4.837435000	-12.577208000	25.350236000
C	3.843679000	-12.371464000	24.442368000
C	4.372786000	-12.736035000	23.151484000
H	4.815289000	-12.425502000	26.421924000
H	2.836044000	-12.014740000	24.613802000
C	3.646789000	-12.694243000	21.969517000
H	2.620000000	-12.347102000	22.024912000
N	5.397316000	-13.518735000	20.463466000
C	4.133445000	-13.062518000	20.724215000
C	3.373499000	-12.988811000	19.498061000
C	4.200810000	-13.396610000	18.498411000
C	5.467837000	-13.720331000	19.111455000
H	2.344120000	-12.660138000	19.432297000
H	3.994144000	-13.473330000	17.438529000
C	6.588146000	-14.144766000	18.413301000
H	6.497904000	-14.254068000	17.337348000
N	8.122170000	-14.361081000	20.320732000
C	7.818278000	-14.436183000	18.985030000
C	8.969878000	-14.890402000	18.245401000
C	9.965511000	-15.096663000	19.150650000
C	9.425156000	-14.771463000	20.447556000
H	8.992629000	-15.032791000	17.172502000
H	10.975704000	-15.444452000	18.976175000
C	10.125075000	-14.892375000	21.639696000
H	11.158354000	-15.219548000	21.583533000
N	8.327905000	-14.195670000	23.162629000
C	9.606976000	-14.616504000	22.896746000
C	10.365334000	-14.688220000	24.121257000
C	9.536792000	-14.281486000	25.123506000
C	8.269961000	-13.960177000	24.513721000
H	11.402668000	-14.991906000	24.183596000
H	9.752562000	-14.181307000	26.179764000
C	7.180468000	-13.443581000	25.200283000
H	7.278071000	-13.316253000	26.273753000
O	6.230299000	-15.494947000	21.983781000
S	7.695277000	-11.699864000	21.459515000
H	6.953135000	-11.055430000	22.387077000
C	6.808881000	-17.683591000	25.011900000
C	7.429270000	-18.490882000	25.961074000
C	8.724765000	-19.171169000	25.624127000
C	8.970168000	-19.256029000	24.145191000
C	8.274397000	-18.413548000	23.283067000
N	7.289913000	-17.593150000	23.741995000
C	8.505369000	-18.333756000	21.793843000
C	5.563600000	-16.869597000	25.264856000
C	9.997843000	-20.194157000	23.639692000
O	10.388375000	-20.281258000	22.487563000
O	10.473372000	-20.998727000	24.627194000
C	11.466113000	-21.966822000	24.223604000
C	11.852230000	-22.766425000	25.454286000
C	6.870497000	-18.644495000	27.323402000
O	5.902412000	-18.055945000	27.775104000
O	7.562751000	-19.561982000	28.049569000
C	7.073793000	-19.801555000	29.387252000
C	7.967773000	-20.852485000	30.019174000
C	9.912677000	-18.400000000	26.307164000
H	8.735232000	-20.176267000	26.056936000
H	6.714144000	-16.078353000	22.619393000
H	9.535171000	-18.047957000	21.563928000

H	8.349025000	-19.308665000	21.322171000
H	7.816960000	-17.604725000	21.363651000
H	5.293938000	-16.334393000	24.353117000
H	4.733519000	-17.514065000	25.570369000
H	5.709937000	-16.157629000	26.081088000
H	11.041283000	-22.600157000	23.438140000
H	12.321180000	-21.437047000	23.791127000
H	12.603836000	-23.517229000	25.187070000
H	12.274725000	-22.117553000	26.228465000
H	10.983302000	-23.283569000	25.874006000
H	6.031202000	-20.130232000	29.327224000
H	7.088733000	-18.858669000	29.943596000
H	7.625452000	-21.066335000	31.037575000
H	9.005325000	-20.506520000	30.070864000
H	7.943058000	-21.784378000	29.445057000
H	10.852938000	-18.916427000	26.098786000
H	9.759045000	-18.372536000	27.388837000
H	9.969240000	-17.376675000	25.923491000

^{2}IM

Fe	6.863194000	-13.846938000	21.807744000
N	5.677353000	-13.141060000	23.279277000
C	5.975946000	-13.071053000	24.617432000
C	4.836116000	-12.577258000	25.349780000
C	3.842925000	-12.370577000	24.441512000
C	4.372497000	-12.734854000	23.150734000
H	4.813517000	-12.426034000	26.421526000
H	2.835390000	-12.013407000	24.612597000
C	3.647119000	-12.692266000	21.968414000
H	2.620494000	-12.344576000	22.023376000
N	5.397980000	-13.517379000	20.463014000
C	4.134223000	-13.060450000	20.723276000
C	3.374954000	-12.985890000	19.496779000
C	4.202516000	-13.393847000	18.497397000
C	5.469043000	-13.718515000	19.110925000
H	2.345806000	-12.656576000	19.430614000
H	3.996326000	-13.470078000	17.437387000
C	6.589423000	-14.143285000	18.413116000
H	6.499601000	-14.252179000	17.337088000
N	8.122381000	-14.360919000	20.321186000
C	7.819138000	-14.435508000	18.985331000
C	8.970848000	-14.890176000	18.246149000
C	9.965903000	-15.097245000	19.151843000
C	9.425074000	-14.772113000	20.448572000
H	8.994053000	-15.032288000	17.173222000
H	10.975986000	-15.445565000	18.977788000
C	10.124359000	-14.893766000	21.640997000
H	11.157498000	-15.221447000	21.585205000
N	8.326845000	-14.196729000	23.163351000
C	9.605843000	-14.618004000	22.897911000
C	10.363653000	-14.690318000	24.122724000
C	9.534825000	-14.283516000	25.124725000
C	8.268387000	-13.961579000	24.514464000
H	11.400848000	-14.994402000	24.185433000
H	9.750176000	-14.183690000	26.181101000
C	7.178761000	-13.444738000	25.200652000
H	7.275865000	-13.317845000	26.274219000
O	6.229768000	-15.494656000	21.983679000
S	7.696850000	-11.699933000	21.461176000
H	6.955300000	-11.055659000	22.389319000
C	6.807235000	-17.685655000	25.011445000
C	7.429009000	-18.491523000	25.960966000
C	8.724570000	-19.171464000	25.623623000
C	8.969351000	-19.256950000	24.144629000
C	8.272181000	-18.415836000	23.282251000
N	7.286709000	-17.596534000	23.740898000
C	8.502432000	-18.336693000	21.792874000
C	5.561743000	-16.872060000	25.264675000
C	9.997459000	-20.194648000	23.639187000
O	10.387719000	-20.281915000	22.486981000
O	10.473715000	-20.998605000	24.626836000
C	11.466903000	-21.966275000	24.223317000
C	11.853572000	-22.765442000	25.454108000
C	6.871275000	-18.644365000	27.323814000
O	5.903329000	-18.055799000	27.775787000
O	7.564321000	-19.561137000	28.050127000
C	7.076395000	-19.799900000	29.388336000
C	7.971035000	-20.850234000	30.020310000
C	9.912558000	-18.399568000	26.305756000
H	8.735654000	-20.176366000	26.056912000
H	6.713308000	-16.078352000	22.618992000
H	9.531824000	-18.049892000	21.562362000
H	8.346942000	-19.312017000	21.321768000
H	7.813032000	-17.608609000	21.362649000
H	5.291266000	-16.337729000	24.352661000
H	4.732139000	-17.516630000	25.571290000
H	5.708344000	-16.159358000	26.080217000
H	11.042301000	-22.599975000	23.438026000
H	12.321651000	-21.436148000	23.790637000
H	12.605524000	-23.515918000	25.186948000
H	12.275830000	-22.116210000	26.228115000
H	10.984953000	-23.282945000	25.874026000
H	6.033822000	-20.128809000	29.329292000
H	7.091570000	-18.856633000	29.944026000
H	7.629512000	-21.063448000	31.039113000
H	9.008564000	-20.504049000	30.070991000
H	7.946059000	-21.782526000	29.446853000
H	10.852895000	-18.915752000	26.097131000
H	9.759441000	-18.371723000	27.387487000

H	9.968527000	-17.376393000	25.921612000

^{4}**TS**2

Fe	6.884226000	-13.837538000	21.807147000
N	5.706833000	-13.120386000	23.280259000
C	6.014350000	-13.038130000	24.615674000
C	4.878993000	-12.538875000	25.351285000
C	3.879634000	-12.340926000	24.447883000
C	4.400964000	-12.715949000	23.156761000
H	4.863359000	-12.378140000	26.421775000
H	2.873001000	-11.982921000	24.622515000
C	3.668015000	-12.683024000	21.978796000
H	2.641431000	-12.335852000	22.037734000
N	5.409891000	-13.517619000	20.468511000
C	4.147448000	-13.060261000	20.733360000
C	3.379789000	-12.996721000	19.511318000
C	4.201047000	-13.412295000	18.509978000
C	5.472027000	-13.730559000	19.117950000
H	2.349822000	-12.669149000	19.449324000
H	3.987884000	-13.497861000	17.452059000
C	6.588000000	-14.160859000	18.416431000
H	6.491070000	-14.279065000	17.341998000
N	8.134217000	-14.360981000	20.315799000
C	7.821918000	-14.446988000	18.982749000
C	8.969717000	-14.904664000	18.239257000
C	9.971770000	-15.101193000	19.139558000
C	9.439115000	-14.766812000	20.437321000
H	8.985841000	-15.055510000	17.167393000
H	10.981673000	-15.447853000	18.961162000
C	10.146714000	-14.876362000	21.625958000
H	11.180710000	-15.200586000	21.565780000
N	8.357215000	-14.173149000	23.155105000
C	9.635602000	-14.592419000	22.884124000
C	10.402283000	-14.651717000	24.104095000
C	9.578951000	-14.239101000	25.108537000
C	8.307267000	-13.926517000	24.504311000
H	11.441412000	-14.950285000	24.162047000
H	9.801895000	-14.128014000	26.162258000
C	7.221596000	-13.405393000	25.193631000
H	7.326288000	-13.268411000	26.265261000
O	6.250870000	-15.482158000	21.996356000
S	7.712791000	-11.691759000	21.436754000
H	6.975870000	-11.040353000	22.363578000
C	6.792233000	-17.697767000	24.990942000
C	7.367050000	-18.584028000	25.922426000
C	8.504745000	-19.333316000	25.519015000
C	8.876173000	-19.341732000	24.147733000
C	8.243212000	-18.427264000	23.283850000
N	7.269231000	-17.609214000	23.732548000
C	8.561136000	-18.298307000	21.817184000
C	5.609784000	-16.814348000	25.291224000
C	9.925621000	-20.274039000	23.666430000
O	10.302356000	-20.393386000	22.514560000
O	10.445992000	-21.016934000	24.678835000
C	11.452076000	-21.980206000	24.296098000
C	11.882581000	-22.715335000	25.551997000
C	6.835371000	-18.723180000	27.300878000
O	5.853273000	-18.159427000	27.748930000
O	7.580829000	-19.580727000	28.046745000
C	7.114145000	-19.806154000	29.395120000
C	8.060850000	-20.797149000	30.046785000
C	10.179791000	-18.150396000	26.437652000
H	8.842375000	-20.142118000	26.151220000
H	6.728808000	-16.075805000	22.628720000
H	9.615157000	-18.054914000	21.655255000
H	8.388890000	-19.246775000	21.299301000
H	7.931996000	-17.521879000	21.379894000
H	5.368686000	-16.222294000	24.407287000
H	4.741820000	-17.415772000	25.578670000
H	5.809681000	-16.152079000	26.138214000
H	11.025297000	-22.657429000	23.549384000
H	12.285541000	-21.451868000	23.821969000
H	12.644617000	-23.461354000	25.301449000
H	12.307827000	-22.023880000	26.286926000
H	11.034731000	-23.231485000	26.013997000
H	6.087617000	-20.184175000	29.353870000
H	7.090338000	-18.848964000	29.925924000
H	7.737743000	-20.997681000	31.074134000
H	9.082012000	-20.403128000	30.078907000
H	8.073814000	-21.745146000	29.499210000
H	11.010179000	-18.782731000	26.142818000
H	9.869864000	-18.198571000	27.475925000
H	10.064070000	-17.202178000	25.922709000

^{2}**TS**2

Fe	6.884338000	-13.837583000	21.807285000
N	5.706992000	-13.120556000	23.280505000
C	6.014387000	-13.038846000	24.615989000
C	4.879088000	-12.539515000	25.351642000
C	3.879887000	-12.340977000	24.448197000
C	4.401262000	-12.715713000	23.157009000
H	4.863381000	-12.379141000	26.422185000
H	2.873336000	-11.982747000	24.622835000
C	3.668468000	-12.682202000	21.978963000
H	2.641978000	-12.334746000	22.037869000
N	5.410307000	-13.516879000	20.468647000
C	4.147960000	-13.059194000	20.733484000
C	3.380486000	-12.994996000	19.511369000
C	4.201742000	-13.410471000	18.509987000
C	5.472544000	-13.729343000	19.117998000

H	2.350630000	-12.667079000	19.449357000
H	3.988688000	-13.495597000	17.452011000
C	6.588454000	-14.159724000	18.416444000
H	6.491605000	-14.277522000	17.341960000
N	8.134347000	-14.360967000	20.315927000
C	7.822211000	-14.446443000	18.982817000
C	8.969941000	-14.904307000	18.239331000
C	9.971789000	-15.101512000	19.139708000
C	9.439078000	-14.767344000	20.437506000
H	8.986154000	-15.054813000	17.167421000
H	10.981584000	-15.448500000	18.961345000
C	10.146497000	-14.877500000	21.626187000
H	11.180392000	-15.202050000	21.566025000
N	8.357075000	-14.174161000	23.155365000
C	9.635367000	-14.593700000	22.884386000
C	10.401958000	-14.653420000	24.104386000
C	9.578654000	-14.240803000	25.108864000
C	8.307084000	-13.927814000	24.504618000
H	11.441011000	-14.952245000	24.162350000
H	9.801563000	-14.129973000	26.162620000
C	7.221464000	-13.406607000	25.193969000
H	7.326075000	-13.269956000	26.265650000
O	6.250834000	-15.482201000	21.996276000
S	7.713890000	-11.691983000	21.437959000
H	6.977291000	-11.040725000	22.365142000
C	6.790955000	-17.699020000	24.990164000
C	7.366441000	-18.584482000	25.922001000
C	8.504365000	-19.333491000	25.518701000
C	8.875613000	-19.342293000	24.147368000
C	8.241978000	-18.428592000	23.283160000
N	7.267437000	-17.611046000	23.731538000
C	8.559657000	-18.300039000	21.816399000
C	5.608189000	-16.815949000	25.290251000
C	9.925345000	-20.274387000	23.666275000
O	10.301765000	-20.394228000	22.514354000
O	10.446391000	-21.016452000	24.678945000
C	11.452765000	-21.979511000	24.296436000
C	11.884166000	-22.713565000	25.552656000
C	6.835043000	-18.723263000	27.300599000
O	5.852752000	-18.159765000	27.748546000
O	7.581023000	-19.580112000	28.046753000
C	7.114652000	-19.805118000	29.395305000
C	8.061929000	-20.795347000	30.047303000
C	10.179040000	-18.149560000	26.436632000
H	8.842415000	-20.141907000	26.151177000
H	6.728573000	-16.076142000	22.628423000
H	9.613560000	-18.056282000	21.654244000
H	8.387709000	-19.248747000	21.298854000
H	7.930144000	-17.523996000	21.378953000
H	5.366754000	-16.224380000	24.406078000
H	4.740497000	-17.417579000	25.578092000
H	5.807963000	-16.153226000	26.136914000
H	11.025978000	-22.657454000	23.550378000
H	12.285772000	-21.451110000	23.821572000
H	12.646421000	-23.459421000	25.302289000
H	12.309436000	-22.021402000	26.286904000
H	11.036755000	-23.229765000	26.015404000
H	6.088299000	-20.183651000	29.354391000
H	7.090466000	-18.847685000	29.925654000
H	7.739072000	-20.995540000	31.074797000
H	9.082907000	-20.400822000	30.079082000
H	8.075266000	-21.743600000	29.500182000
H	11.009737000	-18.781382000	26.141567000
H	9.869561000	-18.197796000	27.475037000
H	10.062427000	-17.201526000	25.921562000

B. Deethylation of DDEP

^{4}RC

Fe	6.861323000	-13.479379000	21.640524000
N	5.549791000	-12.355994000	22.686006000
C	5.783449000	-11.716554000	23.877461000
C	4.566905000	-11.123860000	24.376165000
C	3.591115000	-11.418541000	23.474178000
C	4.216051000	-12.188785000	22.426920000
H	4.487876000	-10.560577000	25.296967000
H	2.543529000	-11.147489000	23.499195000
C	3.550317000	-12.674481000	21.304941000
H	2.488290000	-12.463018000	21.226719000
N	5.455921000	-13.775350000	20.222877000
C	4.129875000	-13.403400000	20.279946000
C	3.422085000	-13.901991000	19.126633000
C	4.322881000	-14.595796000	18.380751000
C	5.586255000	-14.520206000	19.071824000
H	2.369969000	-13.737362000	18.932909000
H	4.164968000	-15.118368000	17.446174000
C	6.755785000	-15.123125000	18.638879000
H	6.710741000	-15.693719000	17.716488000
N	8.226147000	-14.365219000	20.447639000
C	7.987961000	-15.034943000	19.274694000
C	9.205815000	-15.627363000	18.777919000
C	10.187351000	-15.306172000	19.663173000
C	9.567132000	-14.525093000	20.705151000
H	9.281537000	-16.206284000	17.866669000
H	11.237114000	-15.567706000	19.631856000
C	10.233380000	-14.022244000	21.812089000
H	11.297121000	-14.225653000	21.887603000
N	8.331273000	-12.934082000	22.907872000
C	9.650981000	-13.286548000	22.839798000
C	10.369345000	-12.777932000	23.982961000

C	9.461865000	-12.110120000	24.746163000
C	8.194725000	-12.213064000	24.064149000
H	11.427238000	-12.921538000	24.160749000
H	9.617992000	-11.589549000	25.682127000
C	7.012191000	-11.642880000	24.519354000
H	7.051930000	-11.096931000	25.456952000
O	6.510780000	-14.842694000	22.471863000
S	7.327285000	-11.509358000	20.117180000
H	6.154739000	-10.856425000	20.283854000
C	6.673810000	-16.976569000	25.605037000
C	7.003154000	-17.962390000	26.493703000
C	8.172948000	-18.886628000	26.171895000
C	8.234740000	-19.125683000	24.667405000
C	7.869480000	-18.118534000	23.819327000
N	7.259336000	-16.994285000	24.350463000
C	8.012963000	-18.102289000	22.318785000
C	5.669265000	-15.870856000	25.807650000
C	8.769942000	-20.380800000	24.131658000
O	8.915754000	-20.675638000	22.952079000
O	9.116352000	-21.249962000	25.127756000
C	9.634160000	-22.518988000	24.695294000
C	9.960887000	-23.325498000	25.940125000
C	6.239762000	-18.105275000	27.738024000
O	5.362703000	-17.357148000	28.151771000
O	6.597746000	-19.226251000	28.432424000
C	5.872821000	-19.460680000	29.651061000
C	6.380618000	-20.765123000	30.240444000
C	9.534787000	-18.317642000	26.666679000
H	8.013964000	-19.841673000	26.673912000
H	6.999669000	-16.251595000	23.701926000
H	9.025582000	-18.375397000	22.015639000
H	7.350437000	-18.844937000	21.862391000
H	7.760364000	-17.115049000	21.920688000
H	5.730102000	-15.144901000	24.990954000
H	4.652284000	-16.276468000	25.839607000
H	5.823852000	-15.368855000	26.764046000
H	8.886229000	-23.018825000	24.069914000
H	10.519581000	-22.353662000	24.071755000
H	10.357485000	-24.307301000	25.657718000
H	10.710966000	-22.813069000	26.551623000
H	9.065951000	-23.477386000	26.552483000
H	4.800763000	-19.503046000	29.430300000
H	6.030490000	-18.616955000	30.332008000
H	5.851010000	-20.985803000	31.174188000
H	7.452281000	-20.705797000	30.457736000
H	6.217510000	-21.596264000	29.546390000
H	10.317837000	-19.012467000	26.335187000
H	9.722876000	-17.365609000	26.150956000
C	9.649821000	-18.110988000	28.180544000
H	9.475356000	-19.045818000	28.724602000
H	10.650197000	-17.746663000	28.444713000
H	8.924414000	-17.375183000	28.544939000

^{2}RC

Fe	6.881967000	-13.464218000	21.651087000
N	5.582752000	-12.336430000	22.702591000
C	5.832082000	-11.682427000	23.882881000
C	4.620799000	-11.091347000	24.395450000
C	3.631938000	-11.401403000	23.513073000
C	4.243235000	-12.180294000	22.464281000
H	4.553920000	-10.518087000	25.311033000
H	2.583375000	-11.135934000	23.552624000
C	3.558858000	-12.685463000	21.362471000
H	2.493800000	-12.483098000	21.303789000
N	5.450387000	-13.784533000	20.256485000
C	4.122012000	-13.426866000	20.337190000
C	3.393600000	-13.955237000	19.210190000
C	4.284673000	-14.655304000	18.458420000
C	5.562370000	-14.553232000	19.119295000
H	2.335689000	-13.805376000	19.037145000
H	4.111159000	-15.198577000	17.538523000
C	6.728155000	-15.153446000	18.672736000
H	6.668938000	-15.741615000	17.762258000
N	8.230355000	-14.352775000	20.437196000
C	7.972256000	-15.044810000	19.281501000
C	9.184065000	-15.637223000	18.769636000
C	10.181481000	-15.293739000	19.628361000
C	9.576831000	-14.499319000	20.669445000
H	9.245044000	-16.231509000	17.867209000
H	11.232252000	-15.548428000	19.579634000
C	10.260734000	-13.971802000	21.753948000
H	11.327776000	-14.163546000	21.811047000
N	8.368983000	-12.888556000	22.875165000
C	9.691913000	-13.226105000	22.781731000
C	10.429133000	-12.693619000	23.901319000
C	9.530695000	-12.028274000	24.677374000
C	8.250076000	-12.155179000	24.026204000
H	11.492670000	-12.821180000	24.056193000
H	9.701607000	-11.493840000	25.602873000
C	7.071659000	-11.593021000	24.501156000
H	7.125097000	-11.037401000	25.432338000
O	6.542227000	-14.832688000	22.474141000
S	7.289612000	-11.509113000	20.063199000
H	6.114499000	-10.866168000	20.249847000
C	6.657417000	-16.994076000	25.593529000
C	6.977686000	-17.982073000	26.482946000
C	8.159875000	-18.896337000	26.177311000
C	8.247862000	-19.128563000	24.672854000
C	7.891540000	-18.119369000	23.823586000

N	7.265567000	-17.001327000	24.349528000
C	8.060679000	-18.094535000	22.325859000
C	5.641624000	-15.896418000	25.783594000
C	8.798136000	-20.378369000	24.140356000
O	8.962365000	-20.668409000	22.961927000
O	9.135838000	-21.249019000	25.138286000
C	9.667012000	-22.513422000	24.708805000
C	9.982135000	-23.321905000	25.955363000
C	6.193266000	-18.136566000	27.712514000
O	5.301844000	-17.398243000	28.113133000
O	6.549205000	-19.256650000	28.409715000
C	5.805093000	-19.501476000	29.614523000
C	6.314659000	-20.802851000	30.209183000
C	9.508861000	-18.319422000	26.696729000
H	7.999422000	-19.854646000	26.672580000
H	7.012862000	-16.257132000	23.700309000
H	9.076637000	-18.371612000	22.038352000
H	7.401460000	-18.830180000	21.853354000
H	7.820150000	-17.103300000	21.930075000
H	5.713124000	-15.164995000	24.972577000
H	4.626976000	-16.308971000	25.793652000
H	5.774514000	-15.399219000	26.745735000
H	8.930525000	-23.015849000	24.071989000
H	10.559611000	-22.340861000	24.097575000
H	10.388346000	-24.300445000	25.675272000
H	10.720960000	-22.806874000	26.578272000
H	9.080146000	-23.481026000	26.555423000
H	4.737507000	-19.553223000	29.374832000
H	5.942730000	-18.658397000	30.300618000
H	5.771356000	-21.030870000	31.133258000
H	7.381905000	-20.734307000	30.444741000
H	6.171009000	-21.633602000	29.510371000
H	10.302692000	-19.006357000	26.374505000
H	9.697834000	-17.363358000	26.188863000
C	9.597972000	-18.119574000	28.213268000
H	9.423175000	-19.058751000	28.749707000
H	10.590724000	-17.747841000	28.495319000
H	8.860292000	-17.392144000	28.569745000

^{4}**TS**1

Fe	6.720000000	-13.826047000	21.847703000
N	5.399358000	-12.632325000	22.799225000
C	5.479550000	-12.176616000	24.090228000
C	4.295740000	-11.419997000	24.421479000
C	3.499022000	-11.434454000	23.317907000
C	4.194634000	-12.200276000	22.311132000
H	4.114454000	-10.949569000	25.379581000
H	2.526927000	-10.978064000	23.180995000
C	3.693774000	-12.462401000	21.042096000
H	2.720504000	-12.049636000	20.795552000
N	5.561994000	-13.803137000	20.195478000
C	4.335195000	-13.213343000	20.066962000
C	3.795970000	-13.471181000	18.750501000
C	4.718499000	-14.220972000	18.090523000
C	5.821962000	-14.419507000	19.003253000
H	2.837294000	-13.113033000	18.397347000
H	4.678296000	-14.607897000	17.080224000
C	6.982747000	-15.113867000	18.689516000
H	7.056833000	-15.540801000	17.694217000
N	8.147250000	-14.834233000	20.831935000
C	8.063781000	-15.293520000	19.543286000
C	9.271200000	-16.001403000	19.192272000
C	10.081776000	-15.962833000	20.285782000
C	9.369195000	-15.233669000	21.307548000
H	9.458071000	-16.459292000	18.229270000
H	11.072671000	-16.381987000	20.406298000
C	9.859716000	-14.995424000	22.585696000
H	10.853669000	-15.366999000	22.814773000
N	7.935409000	-13.759153000	23.475639000
C	9.190688000	-14.301316000	23.584576000
C	9.735631000	-14.024053000	24.892943000
C	8.797832000	-13.301181000	25.565445000
C	7.680268000	-13.131106000	24.667641000
H	10.717053000	-14.334376000	25.228869000
H	8.847289000	-12.899244000	26.569526000
C	6.536030000	-12.401516000	24.964210000
H	6.466675000	-11.961548000	25.954316000
O	5.951818000	-15.241371000	22.393228000
S	7.878890000	-11.895056000	21.067991000
H	7.314155000	-10.984061000	21.891811000
C	6.338372000	-16.884048000	25.300168000
C	6.945662000	-17.706791000	26.233393000
C	8.092757000	-18.582257000	25.807873000
C	8.028339000	-18.923211000	24.342187000
C	7.369486000	-18.069040000	23.472266000
N	6.641746000	-17.025775000	23.973688000
C	7.320662000	-18.214184000	21.972935000
C	5.276188000	-15.856390000	25.598678000
C	8.728173000	-20.125373000	23.841418000
O	8.794844000	-20.487180000	22.678070000
O	9.304001000	-20.828181000	24.852147000
C	9.990283000	-22.037646000	24.462563000
C	10.557887000	-22.666523000	25.721606000
C	6.482242000	-17.718419000	27.637415000
O	5.749962000	-16.893351000	28.159245000
O	6.950283000	-18.800359000	28.312868000
C	6.533474000	-18.905474000	29.691209000
C	7.108014000	-20.196029000	30.245695000
C	9.483528000	-17.869493000	26.076944000

H	8.098894000	-19.498459000	26.402275000
H	6.300854000	-16.110794000	23.180583000
H	8.324322000	-18.255264000	21.543807000
H	6.834246000	-19.156512000	21.702351000
H	6.767794000	-17.381392000	21.536167000
H	4.969544000	-15.357817000	24.677918000
H	4.406719000	-16.331975000	26.063322000
H	5.638007000	-15.115789000	26.314589000
H	9.278789000	-22.698625000	23.957048000
H	10.772296000	-21.783311000	23.739789000
H	11.087320000	-23.591319000	25.467648000
H	11.264070000	-21.990216000	26.214245000
H	9.760931000	-22.910311000	26.431660000
H	5.439691000	-18.891252000	29.730823000
H	6.893030000	-18.026713000	30.236845000
H	6.808939000	-20.315081000	31.292804000
H	8.202004000	-20.191811000	30.199386000
H	6.742088000	-21.060813000	29.682752000
H	10.256304000	-18.537897000	25.682522000
H	9.507519000	-16.950302000	25.480067000
C	9.787005000	-17.548960000	27.541797000
H	9.742342000	-18.445179000	28.168973000
H	10.796620000	-17.129058000	27.624194000
H	9.091653000	-16.810396000	27.954158000

^{2}**TS**1

Fe	6.672687000	-13.669465000	21.968692000
N	5.177419000	-12.442488000	22.534098000
C	5.091490000	-11.728691000	23.702135000
C	3.819897000	-11.051253000	23.776060000
C	3.134054000	-11.379559000	22.646864000
C	3.989188000	-12.251236000	21.878154000
H	3.507408000	-10.417508000	24.596249000
H	2.141472000	-11.069721000	22.345613000
C	3.643601000	-12.800811000	20.650151000
H	2.664373000	-12.553520000	20.252113000
N	5.716661000	-14.031821000	20.221581000
C	4.459221000	-13.618036000	19.879924000
C	4.106888000	-14.123198000	18.571663000
C	5.175961000	-14.836089000	18.127774000
C	6.177615000	-14.773017000	19.168229000
H	3.164165000	-13.936370000	18.073293000
H	5.296451000	-15.360899000	17.188618000
C	7.424345000	-15.377943000	19.094733000
H	7.661130000	-15.929498000	18.190263000
N	8.266983000	-14.709257000	21.302310000
C	8.389840000	-15.344690000	20.093390000
C	9.662438000	-16.018780000	20.017313000
C	10.298821000	-15.792392000	21.200438000
C	9.420156000	-14.970013000	21.995482000
H	10.006518000	-16.588944000	19.163841000
H	11.275916000	-16.134699000	21.516870000
C	9.720073000	-14.507182000	23.271651000
H	10.680481000	-14.791701000	23.690642000
N	7.684039000	-13.199560000	23.661175000
C	8.916978000	-13.664301000	24.029967000
C	9.291420000	-13.115175000	25.312747000
C	8.272849000	-12.300711000	25.702245000
C	7.271659000	-12.362023000	24.662818000
H	10.222786000	-13.323362000	25.823933000
H	8.190614000	-11.704635000	26.602211000
C	6.066697000	-11.673450000	24.690072000
H	5.864719000	-11.051806000	25.556829000
O	5.946017000	-15.031770000	22.697418000
S	7.760931000	-11.814259000	20.902519000
H	6.896347000	-10.844808000	21.276805000
C	6.777093000	-16.702184000	25.497951000
C	7.324986000	-17.673532000	26.318586000
C	8.180227000	-18.752940000	25.714158000
C	7.823057000	-19.010344000	24.273533000
C	7.245376000	-17.999157000	23.524571000
N	6.869890000	-16.833590000	24.138116000
C	6.955674000	-18.067814000	22.047080000
C	6.020364000	-15.488805000	25.974636000
C	8.187906000	-20.301698000	23.655166000
O	8.043690000	-20.608254000	22.482803000
O	8.713494000	-21.162889000	24.567341000
C	9.079065000	-22.464433000	24.060806000
C	9.636198000	-23.266750000	25.222134000
C	7.107518000	-17.634488000	27.780657000
O	6.701909000	-16.679674000	28.423188000
O	7.392791000	-18.829370000	28.361977000
C	7.187132000	-18.894535000	29.789562000
C	7.514404000	-20.307839000	30.235168000
C	9.713142000	-18.357694000	25.769154000
H	8.078896000	-19.672957000	26.293483000
H	6.443985000	-15.890420000	23.414296000
H	7.879396000	-18.192805000	21.474911000
H	6.337068000	-18.940345000	21.821790000
H	6.445966000	-17.160050000	21.722506000
H	5.583039000	-14.961818000	25.126041000
H	5.234483000	-15.778549000	26.676730000
H	6.682092000	-14.809940000	26.520138000
H	8.191847000	-22.934409000	23.624096000
H	9.812122000	-22.338647000	23.257288000
H	9.922795000	-24.266597000	24.878295000
H	10.521994000	-22.781913000	25.645519000
H	8.890382000	-23.376449000	26.016165000
H	6.149163000	-18.624408000	30.008609000

H	7.828522000	-18.150648000	30.273756000
H	7.365374000	-20.399230000	31.316617000
H	8.555591000	-20.560926000	30.009532000
H	6.866619000	-21.035570000	29.735757000
H	10.258758000	-19.154711000	25.252911000
H	9.843928000	-17.440963000	25.181180000
C	10.295502000	-18.170076000	27.171853000
H	10.153311000	-19.064010000	27.787820000
H	11.372332000	-17.975802000	27.101303000
H	9.843887000	-17.321874000	27.697100000

^{4}IM

Fe	6.795684000	-13.724356000	21.560073000
N	5.641666000	-12.682247000	22.844525000
C	5.905719000	-12.423978000	24.166817000
C	4.799546000	-11.713155000	24.758882000
C	3.859087000	-11.565383000	23.785411000
C	4.387909000	-12.182701000	22.594339000
H	4.760389000	-11.388321000	25.790855000
H	2.887240000	-11.093031000	23.851709000
C	3.704271000	-12.267757000	21.389670000
H	2.715651000	-11.822500000	21.342780000
N	5.408534000	-13.485862000	20.116069000
C	4.188382000	-12.879021000	20.242817000
C	3.475624000	-12.938485000	18.987856000
C	4.288118000	-13.579167000	18.104988000
C	5.498985000	-13.912231000	18.818384000
H	2.485587000	-12.532347000	18.823886000
H	4.106102000	-13.809995000	17.063084000
C	6.592973000	-14.545232000	18.248160000
H	6.524052000	-14.820698000	17.200713000
N	8.041453000	-14.587645000	20.232696000
C	7.771530000	-14.850954000	18.913639000
C	8.894002000	-15.524590000	18.309002000
C	9.836573000	-15.672935000	19.280086000
C	9.292349000	-15.092643000	20.482889000
H	8.934696000	-15.841319000	17.274635000
H	10.811960000	-16.137194000	19.209274000
C	9.938845000	-15.086790000	21.710895000
H	10.934811000	-15.515748000	21.754690000
N	8.175408000	-13.986099000	23.019471000
C	9.412566000	-14.564910000	22.883572000
C	10.121462000	-14.508933000	24.138100000
C	9.308442000	-13.866798000	25.023137000
C	8.100355000	-13.529325000	24.311442000
H	11.120383000	-14.895465000	24.296207000
H	9.500339000	-13.617917000	26.059214000
C	7.047422000	-12.808160000	24.856238000
H	7.124577000	-12.517494000	25.899129000
O	6.001102000	-15.256872000	21.964397000
S	7.849575000	-11.753284000	20.912362000
H	7.158339000	-10.897878000	21.697999000
C	6.323614000	-17.077857000	25.260814000
C	6.819976000	-17.851640000	26.306288000
C	8.059962000	-18.665251000	26.088576000
C	8.286434000	-18.983061000	24.638029000
C	7.709325000	-18.171245000	23.666182000
N	6.838786000	-17.180172000	24.005744000
C	7.949438000	-18.308318000	22.182744000
C	5.172404000	-16.109552000	25.388086000
C	9.187523000	-20.099030000	24.274499000
O	9.531614000	-20.407912000	23.145801000
O	9.597826000	-20.792244000	25.370348000
C	10.459404000	-21.921665000	25.111471000
C	10.786347000	-22.562710000	26.447620000
C	6.162647000	-17.854548000	27.633595000
O	5.397108000	-17.005225000	28.057269000
O	6.490367000	-18.960433000	28.351148000
C	5.876798000	-19.066713000	29.654271000
C	6.332396000	-20.377657000	30.268142000
C	9.339330000	-17.876650000	26.609078000
H	8.013664000	-19.585930000	26.675227000
H	6.424318000	-15.779830000	22.690234000
H	9.010359000	-18.212166000	21.937256000
H	7.646260000	-19.299280000	21.831007000
H	7.377766000	-17.543722000	21.654386000
H	4.915957000	-15.723954000	24.400040000
H	4.297796000	-16.592432000	25.833503000
H	5.429083000	-15.275802000	26.047874000
H	9.941192000	-22.613090000	24.439200000
H	11.357171000	-21.570563000	24.592177000
H	11.437384000	-23.430070000	26.293085000
H	11.304137000	-21.857371000	27.105890000
H	9.875851000	-22.901345000	26.952588000
H	4.789367000	-19.024012000	29.535844000
H	6.175477000	-18.202873000	30.257399000
H	5.882345000	-20.499179000	31.259495000
H	7.421380000	-20.402556000	30.379989000
H	6.030039000	-21.226553000	29.646411000
H	10.206652000	-18.495832000	26.359159000
H	9.417941000	-16.950107000	26.028406000
C	9.357783000	-17.563299000	28.106698000
H	9.249988000	-18.470453000	28.710278000
H	10.313424000	-17.094643000	28.370269000
H	8.562937000	-16.867377000	28.394958000

^{2}IM

Fe	6.795238000	-13.725761000	21.557922000
N	5.641980000	-12.682120000	22.841774000
C	5.906196000	-12.423450000	24.163996000

C	4.800653000	-11.711284000	24.755611000
C	3.860447000	-11.563019000	23.781975000
C	4.388779000	-12.181435000	22.591270000
H	4.761719000	-11.385942000	25.787432000
H	2.889059000	-11.089678000	23.847943000
C	3.705265000	-12.266410000	21.386524000
H	2.717088000	-11.820213000	21.339202000
N	5.408728000	-13.486720000	20.113838000
C	4.189093000	-12.878590000	20.240077000
C	3.476663000	-12.937651000	18.984950000
C	4.288802000	-13.579236000	18.102417000
C	5.499130000	-13.913352000	18.816170000
H	2.487056000	-12.530603000	18.820634000
H	4.106868000	-13.810068000	17.060500000
C	6.592594000	-14.547485000	18.246276000
H	6.523642000	-14.823054000	17.198857000
N	8.040367000	-14.590898000	20.231185000
C	7.770643000	-14.854317000	18.912150000
C	8.892486000	-15.529519000	18.308121000
C	9.834485000	-15.678779000	19.279615000
C	9.290561000	-15.097402000	20.482028000
H	8.933153000	-15.846631000	17.273870000
H	10.809308000	-16.144299000	19.209324000
C	9.936770000	-15.091864000	21.710169000
H	10.932216000	-15.522000000	21.754307000
N	8.174481000	-13.988570000	23.017988000
C	9.410995000	-14.568735000	22.882534000
C	10.119879000	-14.512655000	24.137072000
C	9.307460000	-13.869063000	25.021620000
C	8.099759000	-13.530873000	24.309600000
H	11.118391000	-14.900110000	24.295491000
H	9.499518000	-13.619678000	26.057545000
C	7.047410000	-12.808365000	24.853785000
H	7.124669000	-12.517163000	25.896521000
O	5.999932000	-15.257788000	21.963628000
S	7.852070000	-11.756193000	20.910558000
H	7.162027000	-10.899915000	21.696265000
C	6.321950000	-17.078572000	25.261255000
C	6.819937000	-17.850324000	26.307532000
C	8.059868000	-18.664004000	26.089855000
C	8.285357000	-18.983630000	24.639543000
C	7.706319000	-18.174072000	23.666959000
N	6.834957000	-17.183408000	24.005592000
C	7.944918000	-18.313416000	22.183479000
C	5.170746000	-16.110239000	25.388441000
C	9.187008000	-20.099381000	24.276832000
O	9.531175000	-20.409008000	23.148362000
O	9.597877000	-20.791465000	25.373215000
C	10.460166000	-21.920527000	25.115097000
C	10.787564000	-22.560397000	26.451696000
C	6.163847000	-17.851471000	27.635436000
O	5.398734000	-17.001568000	28.058705000
O	6.492129000	-18.956481000	28.354105000
C	5.879746000	-19.060978000	29.657948000
C	6.336427000	-20.370781000	30.273439000
C	9.339534000	-17.874761000	26.608629000
H	8.014036000	-19.584006000	26.677639000
H	6.423096000	-15.780646000	22.689337000
H	9.005369000	-18.215585000	21.936585000
H	7.643295000	-19.305578000	21.833810000
H	7.371174000	-17.550786000	21.654524000
H	4.914058000	-15.725154000	24.400259000
H	4.296204000	-16.593057000	25.834148000
H	5.427434000	-15.276291000	26.047934000
H	9.942361000	-22.612750000	24.443340000
H	11.357683000	-21.569210000	24.595521000
H	11.439160000	-23.427448000	26.297778000
H	11.304911000	-21.854242000	27.109439000
H	9.877298000	-22.899254000	26.956930000
H	4.792194000	-19.018943000	29.540379000
H	6.178565000	-18.196062000	30.259446000
H	5.887277000	-20.490976000	31.265363000
H	7.425516000	-20.395047000	30.384387000
H	6.033895000	-21.220758000	29.653272000
H	10.206684000	-18.494287000	26.358978000
H	9.417846000	-16.948934000	26.026786000
C	9.358912000	-17.559554000	28.105851000
H	9.251353000	-18.465939000	28.710626000
H	10.314779000	-17.090704000	28.368251000
H	8.564337000	-16.863161000	28.393722000

^{4}**TS**2

Fe	6.819349000	-13.722932000	21.561770000
N	5.667958000	-12.684976000	22.852458000
C	5.941827000	-12.418431000	24.171082000
C	4.835959000	-11.712400000	24.769421000
C	3.885790000	-11.576336000	23.803677000
C	4.408833000	-12.194941000	22.610669000
H	4.803329000	-11.383037000	25.800187000
H	2.911138000	-11.110745000	23.876385000
C	3.717088000	-12.286786000	21.411054000
H	2.724487000	-11.849763000	21.371124000
N	5.423532000	-13.487811000	20.124509000
C	4.198912000	-12.892383000	20.260205000
C	3.479090000	-12.954993000	19.009274000
C	4.292123000	-13.585771000	18.119879000
C	5.509963000	-13.910375000	18.825545000
H	2.484495000	-12.557309000	18.852279000
H	4.106040000	-13.815496000	17.078446000

C	6.605376000	-14.534344000	18.247999000
H	6.533153000	-14.806873000	17.200001000
N	8.062854000	-14.576801000	20.226015000
C	7.788640000	-14.835859000	18.907034000
C	8.912279000	-15.500693000	18.294631000
C	9.859822000	-15.647975000	19.260952000
C	9.317655000	-15.075586000	20.468531000
H	8.950231000	-15.812766000	17.258744000
H	10.837445000	-16.106508000	19.183910000
C	9.969310000	-15.070560000	21.693717000
H	10.967442000	-15.495052000	21.731896000
N	8.209041000	-13.976573000	23.012281000
C	9.446493000	-14.552541000	22.869782000
C	10.163031000	-14.492674000	24.119873000
C	9.354511000	-13.848976000	25.008134000
C	8.141377000	-13.515711000	24.303136000
H	11.164625000	-14.874844000	24.272043000
H	9.553247000	-13.595096000	26.041760000
C	7.090978000	-12.794300000	24.852532000
H	7.175333000	-12.498596000	25.893465000
O	6.031260000	-15.257393000	21.965076000
S	7.858578000	-11.744277000	20.908216000
H	7.152967000	-10.890649000	21.682936000
C	6.321007000	-17.084932000	25.247611000
C	6.764594000	-17.934336000	26.278747000
C	7.834218000	-18.829821000	25.995599000
C	8.206069000	-19.042299000	24.635832000
C	7.689517000	-18.167195000	23.663075000
N	6.821911000	-17.188522000	23.999097000
C	8.019090000	-18.253211000	22.195473000
C	5.248149000	-16.041782000	25.423396000
C	9.138811000	-20.138979000	24.282690000
O	9.492794000	-20.446214000	23.158403000
O	9.579182000	-20.804231000	25.385363000
C	10.465670000	-21.915810000	25.132644000
C	10.826559000	-22.526927000	26.474121000
C	6.159160000	-17.902075000	27.632168000
O	5.379737000	-17.063248000	28.049015000
O	6.561884000	-18.955795000	28.390615000
C	6.002358000	-19.021213000	29.719885000
C	6.534253000	-20.281350000	30.377601000
C	9.614651000	-17.726741000	26.720532000
H	8.058829000	-19.604346000	26.715552000
H	6.436853000	-15.789140000	22.697011000
H	9.095650000	-18.174817000	22.020046000
H	7.719406000	-19.224642000	21.790093000
H	7.497897000	-17.457694000	21.661142000
H	5.051272000	-15.561463000	24.463933000
H	4.326266000	-16.490533000	25.806218000
H	5.543655000	-15.288800000	26.159419000
H	9.956422000	-22.631305000	24.479055000
H	11.347375000	-21.551139000	24.595786000
H	11.496288000	-23.380806000	26.324383000
H	11.336723000	-21.799070000	27.113787000
H	9.931796000	-22.879171000	26.997606000
H	4.910540000	-19.028094000	29.642722000
H	6.286596000	-18.118041000	30.270069000
H	6.128081000	-20.371000000	31.391055000
H	7.626986000	-20.258786000	30.446770000
H	6.243338000	-21.171297000	29.810177000
H	10.311138000	-18.531791000	26.501125000
H	9.589341000	-16.931614000	25.978638000
C	9.451426000	-17.330370000	28.154594000
H	9.294030000	-18.196267000	28.806032000
H	10.359882000	-16.816388000	28.510971000
H	8.617847000	-16.633505000	28.294855000

^{2}**TS**2

Fe	6.819393000	-13.722890000	21.561967000
N	5.667945000	-12.684717000	22.852425000
C	5.941274000	-12.418843000	24.171310000
C	4.835482000	-11.712428000	24.769343000
C	3.885910000	-11.575435000	23.803148000
C	4.409236000	-12.193885000	22.610182000
H	4.802495000	-11.383454000	25.800222000
H	2.911485000	-11.109318000	23.875517000
C	3.718050000	-12.284977000	21.410186000
H	2.725732000	-11.847348000	21.369854000
N	5.424422000	-13.486794000	20.124235000
C	4.200089000	-12.890610000	20.259460000
C	3.480834000	-12.952509000	19.008179000
C	4.293889000	-13.583610000	18.119036000
C	5.511196000	-13.909113000	18.825189000
H	2.486561000	-12.554171000	18.850798000
H	4.108154000	-13.812996000	17.077466000
C	6.606486000	-14.533582000	18.247975000
H	6.534550000	-14.805865000	17.199893000
N	8.063068000	-14.577125000	20.226587000
C	7.789305000	-14.835855000	18.907464000
C	8.912848000	-15.501214000	18.295451000
C	9.859884000	-15.649149000	19.262166000
C	9.317496000	-15.076642000	20.469591000
H	8.951079000	-15.813160000	17.259536000
H	10.837289000	-16.108206000	19.185489000
C	9.968638000	-15.072143000	21.695041000
H	10.966553000	-15.497115000	21.733553000
N	8.208314000	-13.977687000	23.013138000
C	9.445593000	-14.554088000	22.871000000
C	10.161678000	-14.494732000	24.121369000

C	9.353013000	-13.850982000	25.009479000
C	8.140274000	-13.517154000	24.304079000
H	11.163091000	-14.877257000	24.273839000
H	9.551429000	-13.597413000	26.043243000
C	7.089884000	-12.795508000	24.853212000
H	7.173848000	-12.500197000	25.894288000
O	6.030793000	-15.257191000	21.965054000
S	7.860350000	-11.744692000	20.909747000
H	7.155508000	-10.890978000	21.685072000
C	6.319066000	-17.086400000	25.247248000
C	6.763793000	-17.934970000	26.278591000
C	7.833663000	-18.830097000	25.995249000
C	8.204973000	-19.042937000	24.635390000
C	7.687250000	-18.168685000	23.662476000
N	6.818989000	-17.190571000	23.998399000
C	8.016076000	-18.255181000	22.194730000
C	5.245810000	-16.043669000	25.423137000
C	9.138085000	-20.139301000	24.282232000
O	9.491525000	-20.446920000	23.157879000
O	9.579528000	-20.803742000	25.384967000
C	10.466521000	-21.914918000	25.132250000
C	10.828993000	-22.524852000	26.473839000
C	6.159059000	-17.902403000	27.632321000
O	5.379464000	-17.063754000	28.049205000
O	6.562693000	-18.955580000	28.391035000
C	6.003964000	-19.020608000	29.720659000
C	6.536821000	-20.280177000	30.378686000
C	9.614016000	-17.725949000	26.719025000
H	8.059022000	-19.604211000	26.715417000
H	6.435949000	-15.789323000	22.696846000
H	9.092497000	-18.176261000	22.018689000
H	7.716708000	-19.226950000	21.789922000
H	7.494153000	-17.460161000	21.660361000
H	5.048149000	-15.563987000	24.463512000
H	4.324364000	-16.492622000	25.806776000
H	5.541429000	-15.290133000	26.158549000
H	9.957163000	-22.631211000	24.479618000
H	11.347509000	-21.550050000	24.594354000
H	11.499143000	-23.378403000	26.324115000
H	11.339250000	-21.796209000	27.112534000
H	9.934925000	-22.877307000	26.998369000
H	4.912106000	-19.028028000	29.644129000
H	6.288105000	-18.117036000	30.270236000
H	6.131278000	-20.369513000	31.392419000
H	7.629583000	-20.257074000	30.447209000
H	6.245988000	-21.170538000	29.811870000
H	10.310753000	-18.530760000	26.499530000
H	9.587816000	-16.931132000	25.976842000
C	9.451388000	-17.329140000	28.153033000
H	9.294747000	-18.194870000	28.804876000
H	10.359807000	-16.814627000	28.508737000
H	8.617573000	-16.632597000	28.293490000

^{4}PC

Fe	6.821896000	-13.875913000	21.532650000
N	5.768333000	-12.667434000	22.755123000
C	5.985146000	-12.457914000	24.094623000
C	4.966491000	-11.587153000	24.626712000
C	4.124334000	-11.290747000	23.598752000
C	4.625951000	-11.977755000	22.434384000
H	4.913110000	-11.263946000	25.658551000
H	3.235414000	-10.672961000	23.610944000
C	4.015573000	-11.949076000	21.188677000
H	3.114034000	-11.353564000	21.086597000
N	5.581671000	-13.419867000	20.008476000
C	4.468278000	-12.626527000	20.066556000
C	3.832687000	-12.572813000	18.770534000
C	4.584286000	-13.336565000	17.932890000
C	5.680945000	-13.857616000	18.714982000
H	2.931878000	-12.013929000	18.551004000
H	4.431361000	-13.536858000	16.880061000
C	6.692875000	-14.657335000	18.206406000
H	6.645216000	-14.919625000	17.154394000
N	7.996026000	-14.921320000	20.273483000
C	7.768151000	-15.141258000	18.938156000
C	8.810375000	-15.975651000	18.394721000
C	9.662487000	-16.261957000	19.417730000
C	9.141413000	-15.606781000	20.591728000
H	8.864266000	-16.295329000	17.361890000
H	10.560796000	-16.865829000	19.399079000
C	9.706881000	-15.699503000	21.855942000
H	10.622719000	-16.273622000	21.953951000
N	8.056533000	-14.343025000	23.067797000
C	9.196791000	-15.104811000	23.000491000
C	9.825324000	-15.163783000	24.296890000
C	9.068721000	-14.406634000	25.138927000
C	7.974151000	-13.883133000	24.358373000
H	10.736591000	-15.707124000	24.512591000
H	9.226424000	-14.203450000	26.190875000
C	7.011745000	-13.011109000	24.846924000
H	7.067611000	-12.739027000	25.896134000
O	5.771662000	-15.265330000	21.874530000
S	8.209042000	-12.091636000	20.965614000
H	7.665404000	-11.159168000	21.779281000
C	6.210079000	-17.506444000	24.923773000
C	6.759252000	-18.479650000	25.792209000
C	7.459711000	-19.542829000	25.220475000
C	7.610373000	-19.644580000	23.836792000
C	7.054098000	-18.620701000	23.032761000

N	6.394899000	-17.593073000	23.595539000
C	7.129168000	-18.596366000	21.529923000
C	5.385471000	-16.335753000	25.386755000
C	8.347644000	-20.799052000	23.246183000
O	8.564766000	-20.967418000	22.061309000
O	8.765947000	-21.668949000	24.195480000
C	9.493226000	-22.822085000	23.715988000
C	9.872051000	-23.656652000	24.925291000
C	6.624820000	-18.378935000	27.273890000
O	6.028247000	-17.498450000	27.867543000
O	7.262603000	-19.386458000	27.908876000
C	7.236634000	-19.342990000	29.354908000
C	7.916083000	-20.602354000	29.859277000
C	8.867934000	-15.541000000	29.148705000
H	7.888493000	-20.302568000	25.859456000
H	6.126074000	-15.937567000	22.505574000
H	8.166589000	-18.573612000	21.184566000
H	6.690680000	-19.504253000	21.104038000
H	6.599149000	-17.718401000	21.157536000
H	5.037421000	-15.775283000	24.517703000
H	4.530286000	-16.672072000	25.980453000
H	5.966051000	-15.676367000	26.037919000
H	8.856226000	-23.373860000	23.017351000
H	10.371594000	-22.478435000	23.160348000
H	10.425969000	-24.545117000	24.603146000
H	10.505776000	-23.086465000	25.612304000
H	8.980703000	-23.985463000	25.469351000
H	6.196471000	-19.272824000	29.686950000
H	7.755747000	-18.435939000	29.682325000
H	7.923790000	-20.604603000	30.954691000
H	8.951751000	-20.656845000	29.508407000
H	7.386967000	-21.497682000	29.516986000
H	9.848546000	-15.358774000	29.578593000
H	8.829900000	-15.967664000	28.151199000
C	7.631605000	-15.025225000	29.801120000
H	7.698652000	-15.071713000	30.895792000
H	7.443131000	-13.965041000	29.550904000
H	6.750642000	-15.590386000	29.478927000

^{2}PC

Fe	6.838264000	-13.748449000	21.480340000
N	5.745721000	-12.871363000	22.931699000
C	6.089453000	-12.731863000	24.253509000
C	5.009450000	-12.110148000	24.978709000
C	4.004832000	-11.896517000	24.084399000
C	4.468353000	-12.382510000	22.808744000
H	5.030473000	-11.884452000	26.037329000
H	3.030001000	-11.458294000	24.256547000
C	3.713854000	-12.363205000	21.644212000
H	2.715007000	-11.942642000	21.702156000
N	5.363465000	-13.393095000	20.150955000
C	4.140432000	-12.836818000	20.412506000
C	3.355096000	-12.781370000	19.201606000
C	4.126511000	-13.301549000	18.209437000
C	5.384291000	-13.676774000	18.812155000
H	2.348921000	-12.386722000	19.139536000
H	3.887269000	-13.424442000	17.160810000
C	6.452739000	-14.222172000	18.116585000
H	6.326271000	-14.388582000	17.051650000
N	8.016722000	-14.439157000	19.999493000
C	7.672438000	-14.571352000	18.678195000
C	8.765932000	-15.158053000	17.943568000
C	9.766033000	-15.387337000	18.838303000
C	9.286753000	-14.943020000	20.123730000
H	8.749354000	-15.366410000	16.881388000
H	10.741519000	-15.823224000	18.664071000
C	10.007085000	-15.043979000	21.305640000
H	11.011396000	-15.451301000	21.247258000
N	8.307793000	-14.121849000	22.821894000
C	9.545663000	-14.651860000	22.553729000
C	10.332653000	-14.698731000	23.761325000
C	9.566279000	-14.165559000	24.753068000
C	8.308041000	-13.792887000	24.154654000
H	11.345997000	-15.075326000	23.819656000
H	9.821085000	-14.010705000	25.793897000
C	7.280115000	-13.151874000	24.830650000
H	7.419155000	-12.958789000	25.889646000
O	6.091211000	-15.333060000	21.762277000
S	7.813246000	-11.694812000	20.981754000
H	7.124097000	-10.935128000	21.861786000
C	6.104335000	-17.260363000	24.993532000
C	6.471264000	-18.098091000	26.073892000
C	7.495797000	-19.022711000	25.864946000
C	8.126606000	-19.129414000	24.624356000
C	7.710227000	-18.254778000	23.592993000
N	6.742613000	-17.348253000	23.813694000
C	8.280801000	-18.265193000	22.200321000
C	4.997051000	-16.243166000	25.057135000
C	9.205563000	-20.137009000	24.414451000
O	9.770019000	-20.359186000	23.360295000
O	9.505946000	-20.802839000	25.555787000
C	10.522474000	-21.824223000	25.438378000
C	10.695539000	-22.454943000	26.807473000
C	5.797414000	-18.010599000	27.401162000
O	4.861936000	-17.282743000	27.673260000
O	6.352820000	-18.851560000	28.308088000
C	5.741600000	-18.854130000	29.618491000
C	6.487167000	-19.866274000	30.468066000
C	10.416288000	-17.474135000	27.280713000

H	7.798279000	-19.673812000	26.673189000
H	6.478543000	-15.880669000	22.487873000
H	9.355727000	-18.061671000	22.209904000
H	8.165323000	-19.250934000	21.739937000
H	7.770797000	-17.513057000	21.596804000
H	4.892673000	-15.763002000	24.083250000
H	4.052308000	-16.715694000	25.341977000
H	5.195527000	-15.485556000	25.821153000
H	10.202751000	-22.552841000	24.686611000
H	11.446336000	-21.361392000	25.077476000
H	11.459572000	-23.238493000	26.759775000
H	11.012039000	-21.710855000	27.545693000
H	9.759888000	-22.907184000	27.151991000
H	4.682181000	-19.107392000	29.510658000
H	5.798720000	-17.843680000	30.035293000
H	6.049290000	-19.900519000	31.471633000
H	7.544136000	-19.596962000	30.563202000
H	6.423666000	-20.867911000	30.030673000
H	10.714693000	-18.469359000	26.965878000
H	10.269192000	-16.729777000	26.504265000
C	9.950549000	-17.232357000	28.675598000
H	8.912083000	-17.578896000	28.825197000
H	10.564440000	-17.770843000	29.409797000
H	9.969977000	-16.167357000	28.935688000

C. Deisopropylation of DDIP

^{4}**RC**

Fe	-0.086638000	-0.202846000	-0.108776000
N	0.049250000	-0.273467000	1.904309000
C	1.159902000	-0.599659000	2.641782000
C	0.830000000	-0.665024000	4.044119000
C	-0.497085000	-0.380147000	4.146371000
C	-0.972141000	-0.138908000	2.806051000
H	1.533180000	-0.900769000	4.832389000
H	-1.111022000	-0.331665000	5.036442000
C	-2.282926000	0.203830000	2.486703000
H	-2.991058000	0.278520000	3.306289000
N	-2.006511000	0.396775000	0.057407000
C	-2.757791000	0.461214000	1.211607000
C	-4.121040000	0.812768000	0.900093000
C	-4.196519000	0.940063000	-0.451747000
C	-2.879222000	0.671000000	-0.972535000
H	-4.906806000	0.935705000	1.634312000
H	-5.057024000	1.190581000	-1.058582000
C	-2.550587000	0.674547000	-2.317930000
H	-3.344643000	0.895231000	-3.024458000
N	-0.168184000	0.144226000	-2.094604000
C	-1.282128000	0.443353000	-2.835859000
C	-0.951363000	0.503162000	-4.238805000
C	0.379077000	0.238648000	-4.339304000
C	0.858837000	0.009813000	-2.998347000
H	-1.657197000	0.725469000	-5.028577000
H	0.994025000	0.195640000	-5.228855000
C	2.168595000	-0.311110000	-2.676938000
H	2.878422000	-0.384746000	-3.494982000
N	1.901003000	-0.505160000	-0.249076000
C	2.648140000	-0.555266000	-1.393633000
C	4.019544000	-0.882726000	-1.088194000
C	4.091731000	-1.030698000	0.263031000
C	2.764624000	-0.791314000	0.775126000
H	4.808360000	-0.981770000	-1.822391000
H	4.952335000	-1.277890000	0.870806000
C	2.425693000	-0.834456000	2.121929000
H	3.215737000	-1.077154000	2.825885000
O	-0.443254000	-1.792201000	-0.242832000
S	0.213987000	2.315394000	-0.027619000
H	-0.226133000	2.514869000	1.235372000
C	1.594046000	-4.997683000	0.208698000
C	2.462440000	-6.017426000	-0.069989000
C	3.086916000	-6.072920000	-1.460434000
C	2.020608000	-5.681837000	-2.478964000
C	1.158909000	-4.669238000	-2.157573000
N	1.131152000	-4.227235000	-0.845059000
C	0.153161000	-4.006627000	-3.064063000
C	1.001013000	-4.646430000	1.549094000
C	1.915999000	-6.347939000	-3.781557000
O	1.174251000	-6.038258000	-4.705449000
O	2.758584000	-7.418379000	-3.878385000
C	2.698229000	-8.149285000	-5.114609000
C	3.671679000	-9.309866000	-5.004733000
C	2.782993000	-7.009555000	0.961480000
O	2.419672000	-6.994566000	2.130957000
O	3.557533000	-8.023247000	0.473940000
C	3.893978000	-9.057344000	1.413876000
C	4.721298000	-10.093883000	0.673741000
C	4.347191000	-5.142862000	-1.561482000
H	3.413014000	-7.093221000	-1.662101000
H	0.523694000	-3.438066000	-0.624054000
H	0.604135000	-3.719550000	-4.015280000
H	-0.657145000	-4.701623000	-3.309865000
H	-0.277481000	-3.126342000	-2.576947000
H	0.498612000	-3.675487000	1.500401000
H	0.268530000	-5.402133000	1.854275000
H	1.763695000	-4.632183000	2.328852000
H	1.671225000	-8.492225000	-5.280461000
H	2.954099000	-7.479048000	-5.942650000
H	3.656126000	-9.901374000	-5.927151000
H	4.693254000	-8.949912000	-4.843203000
H	3.403662000	-9.965058000	-4.169347000

H	2.972455000	-9.486340000	1.822142000
H	4.445839000	-8.617245000	2.251761000
H	4.995176000	-10.909496000	1.352509000
H	5.641457000	-9.650918000	0.278279000
H	4.157164000	-10.516321000	-0.164257000
H	4.005563000	-4.118650000	-1.349916000
C	5.419721000	-5.501343000	-0.521283000
H	5.789087000	-6.523232000	-0.670240000
H	6.276143000	-4.820231000	-0.604798000
H	5.038070000	-5.429723000	0.502304000
C	4.963964000	-5.150732000	-2.968777000
H	5.306035000	-6.155530000	-3.244400000
H	4.251020000	-4.820031000	-3.731019000
H	5.830801000	-4.478816000	-3.011243000

^{2}RC

Fe	-0.068576000	-0.216036000	-0.108097000
N	0.078023000	-0.274639000	1.902351000
C	1.190758000	-0.602737000	2.635509000
C	0.868661000	-0.659037000	4.039850000
C	-0.456253000	-0.366334000	4.148013000
C	-0.938239000	-0.130736000	2.809243000
H	1.575135000	-0.894020000	4.825387000
H	-1.064843000	-0.309792000	5.041278000
C	-2.251043000	0.210428000	2.497125000
H	-2.953296000	0.288495000	3.321456000
N	-1.991097000	0.392496000	0.066255000
C	-2.736420000	0.456800000	1.223671000
C	-4.104543000	0.794750000	0.917906000
C	-4.188846000	0.910894000	-0.434471000
C	-2.872003000	0.649379000	-0.960695000
H	-4.887218000	0.916218000	1.655664000
H	-5.055029000	1.149189000	-1.038161000
C	-2.550297000	0.646897000	-2.307939000
H	-3.349504000	0.858005000	-3.011591000
N	-0.163272000	0.129268000	-2.096077000
C	-1.283871000	0.418719000	-2.831649000
C	-0.962143000	0.471955000	-4.237114000
C	0.368755000	0.212753000	-4.344802000
C	0.857663000	-0.006924000	-3.005591000
H	-1.674003000	0.686608000	-5.023598000
H	0.978126000	0.167022000	-5.238041000
C	2.169970000	-0.322981000	-2.689492000
H	2.876458000	-0.397029000	-3.510367000
N	1.913010000	-0.516594000	-0.259211000
C	2.655406000	-0.563362000	-1.408009000
C	4.028989000	-0.886243000	-1.109132000
C	4.108127000	-1.036180000	0.241518000
C	2.783350000	-0.801358000	0.760533000
H	4.814667000	-0.981463000	-1.847185000
H	4.972565000	-1.280967000	0.844797000
C	2.452463000	-0.844388000	2.109067000
H	3.246054000	-1.088128000	2.808614000
O	-0.463677000	-1.794393000	-0.243492000
S	0.212887000	2.321652000	-0.046338000
H	-0.237256000	2.526309000	1.212327000
C	1.564805000	-5.000039000	0.205810000
C	2.437712000	-6.016762000	-0.069932000
C	3.073960000	-6.064979000	-1.455416000
C	2.014906000	-5.672471000	-2.481049000
C	1.148025000	-4.663343000	-2.162616000
N	1.108632000	-4.226593000	-0.848675000
C	0.147222000	-4.000291000	-3.074214000
C	0.959942000	-4.655476000	1.542679000
C	1.923033000	-6.333259000	-3.787276000
O	1.187557000	-6.022309000	-4.715769000
O	2.770688000	-7.399892000	-3.882215000
C	2.724220000	-8.124491000	-5.122720000
C	3.703480000	-9.280048000	-5.011387000
C	2.751228000	-7.012810000	0.959837000
O	2.377278000	-7.004075000	2.126023000
O	3.532554000	-8.022559000	0.474747000
C	3.862239000	-9.060412000	1.412877000
C	4.701599000	-10.090028000	0.676647000
C	4.332325000	-5.131230000	-1.542251000
H	3.404318000	-7.083619000	-1.658563000
H	0.498545000	-3.438752000	-0.629673000
H	0.603931000	-3.709858000	-4.021634000
H	-0.660043000	-4.696295000	-3.327159000
H	-0.288567000	-3.122236000	-2.587631000
H	0.455507000	-3.685594000	1.493531000
H	0.227001000	-5.414244000	1.838998000
H	1.716215000	-4.642121000	2.328695000
H	1.700556000	-8.472462000	-5.298501000
H	2.982678000	-7.448364000	-5.945143000
H	3.698559000	-9.866686000	-5.937031000
H	4.721666000	-8.915094000	-4.839959000
H	3.432766000	-9.941236000	-4.181621000
H	2.937751000	-9.494941000	1.808451000
H	4.402983000	-8.622849000	2.259282000
H	4.970531000	-10.908412000	1.354060000
H	5.624672000	-9.641703000	0.294234000
H	4.148643000	-10.509840000	-0.170083000
H	3.986316000	-4.108860000	-1.329274000
C	5.397390000	-5.491171000	-0.494855000
H	5.771232000	-6.511191000	-0.645505000
H	6.252350000	-4.807022000	-0.568096000
H	5.007126000	-5.425582000	0.525893000
C	4.960712000	-5.131218000	-2.944456000

H	5.308430000	-6.133700000	-3.221461000
H	4.252845000	-4.799755000	-3.711086000
H	5.825584000	-4.456203000	-2.977027000

^{4}**TS**1

Fe	0.006122000	-0.009425000	-0.036472000
N	0.034442000	-0.048594000	1.981849000
C	1.139706000	-0.102226000	2.791465000
C	0.736624000	-0.213137000	4.172746000
C	-0.624208000	-0.240595000	4.183042000
C	-1.053916000	-0.141178000	2.808639000
H	1.420088000	-0.267467000	5.010592000
H	-1.291792000	-0.320876000	5.031497000
C	-2.382858000	-0.142524000	2.403954000
H	-3.138789000	-0.217934000	3.179446000
N	-2.019577000	0.061735000	-0.010546000
C	-2.822542000	-0.037868000	1.092108000
C	-4.213773000	0.006161000	0.699975000
C	-4.234425000	0.141265000	-0.652421000
C	-2.855989000	0.174739000	-1.087203000
H	-5.048494000	-0.052811000	1.386824000
H	-5.089582000	0.215658000	-1.312089000
C	-2.452238000	0.312036000	-2.407605000
H	-3.227008000	0.400768000	-3.162683000
N	-0.025488000	0.241931000	-2.036713000
C	-1.132010000	0.345571000	-2.839608000
C	-0.730741000	0.474013000	-4.219068000
C	0.630920000	0.439471000	-4.237597000
C	1.062543000	0.296178000	-2.868635000
H	-1.415001000	0.576486000	-5.051679000
H	1.296219000	0.510755000	-5.088709000
C	2.391936000	0.228010000	-2.469265000
H	3.148141000	0.291833000	-3.245781000
N	2.028837000	0.043185000	-0.051436000
C	2.833072000	0.123957000	-1.155476000
C	4.221930000	0.115392000	-0.759661000
C	4.241346000	0.042337000	0.599759000
C	2.864508000	-0.000495000	1.032958000
H	5.058208000	0.173509000	-1.444776000
H	5.096616000	0.024023000	1.263295000
C	2.459605000	-0.069811000	2.359508000
H	3.234922000	-0.107340000	3.118423000
O	-0.020777000	-1.712513000	-0.137838000
S	-0.014633000	2.374024000	0.052814000
H	0.377278000	2.520029000	1.337925000
C	2.427185000	-3.862638000	-0.965140000
C	3.231060000	-4.677141000	-1.745511000
C	2.982794000	-4.730371000	-3.229799000
C	1.521885000	-4.533428000	-3.535555000
C	0.771235000	-3.720245000	-2.701935000
N	1.309370000	-3.294951000	-1.516610000
C	-0.648468000	-3.281671000	-2.956406000
C	2.640421000	-3.568907000	0.498000000
C	0.912682000	-5.164143000	-4.725388000
O	-0.174393000	-4.890265000	-5.207814000
O	1.705003000	-6.142506000	-5.237055000
C	1.177716000	-6.834595000	-6.389190000
C	2.205804000	-7.867462000	-6.812375000
C	4.337490000	-5.449082000	-1.141310000
O	4.795279000	-5.293408000	-0.020778000
O	4.798110000	-6.410027000	-1.984264000
C	5.866801000	-7.233270000	-1.469950000
C	6.225048000	-8.241132000	-2.546475000
C	3.840028000	-3.583222000	-3.955332000
H	3.327772000	-5.681798000	-3.635609000
H	0.661385000	-2.450920000	-0.827937000
H	-0.711505000	-2.669821000	-3.860583000
H	-1.290099000	-4.149780000	-3.130410000
H	-1.022460000	-2.711852000	-2.105098000
H	1.814902000	-2.970168000	0.884768000
H	2.719564000	-4.499666000	1.066252000
H	3.581567000	-3.034448000	0.653866000
H	0.221819000	-7.293946000	-6.117025000
H	0.976904000	-6.104405000	-7.179948000
H	1.838254000	-8.420920000	-7.683388000
H	3.153433000	-7.390471000	-7.083453000
H	2.398031000	-8.582589000	-6.005969000
H	5.526245000	-7.718289000	-0.549445000
H	6.713920000	-6.590782000	-1.208023000
H	7.030809000	-8.892914000	-2.191441000
H	6.566072000	-7.738685000	-3.457662000
H	5.362717000	-8.866323000	-2.799801000
H	3.479616000	-2.634109000	-3.536388000
C	5.340756000	-3.701660000	-3.655153000
H	5.740937000	-4.664181000	-3.993261000
H	5.884441000	-2.909180000	-4.183317000
H	5.563061000	-3.594938000	-2.589130000
C	3.610543000	-3.557182000	-5.472990000
H	3.897000000	-4.508222000	-5.935780000
H	2.568673000	-3.350248000	-5.735519000
H	4.223901000	-2.767631000	-5.923620000

^{2}**TS**1

Fe	0.008114000	-0.015978000	-0.026390000
N	0.028164000	-0.031306000	1.989555000
C	1.130030000	-0.077426000	2.804940000
C	0.721717000	-0.177411000	4.185040000
C	-0.639218000	-0.206436000	4.190284000
C	-1.063257000	-0.118749000	2.813864000
H	1.401934000	-0.224250000	5.025924000

H	-1.310032000	-0.280727000	5.036704000
C	-2.390361000	-0.124236000	2.403545000
H	-3.149615000	-0.194564000	3.176285000
N	-2.014839000	0.066535000	-0.008969000
C	-2.824330000	-0.027535000	1.088773000
C	-4.213267000	0.020916000	0.690014000
C	-4.226156000	0.155746000	-0.662645000
C	-2.845418000	0.183562000	-1.089618000
H	-5.051755000	-0.033933000	1.372560000
H	-5.077410000	0.233617000	-1.326892000
C	-2.434723000	0.319988000	-2.408199000
H	-3.206102000	0.412380000	-3.166353000
N	-0.010881000	0.233652000	-2.028216000
C	-1.112919000	0.343298000	-2.836099000
C	-0.705692000	0.457735000	-4.214966000
C	0.655463000	0.405561000	-4.228301000
C	1.080023000	0.267605000	-2.856630000
H	-1.385676000	0.562121000	-5.050782000
H	1.324646000	0.461980000	-5.077425000
C	2.407296000	0.189395000	-2.450679000
H	3.166966000	0.236410000	-3.225009000
N	2.033270000	0.040062000	-0.033132000
C	2.842858000	0.100895000	-1.134262000
C	4.230440000	0.093007000	-0.732511000
C	4.243739000	0.039381000	0.627424000
C	2.864412000	0.006523000	1.054666000
H	5.069675000	0.138236000	-1.414908000
H	5.095834000	0.027197000	1.295094000
C	2.452215000	-0.049899000	2.379155000
H	3.223247000	-0.081624000	3.142698000
O	-0.014644000	-1.717158000	-0.128522000
S	0.028077000	2.384195000	0.020681000
H	0.282476000	2.542755000	1.338716000
C	2.427024000	-3.867798000	-0.969883000
C	3.231310000	-4.678759000	-1.752855000
C	2.980342000	-4.730482000	-3.237693000
C	1.515540000	-4.543336000	-3.536209000
C	0.763940000	-3.735765000	-2.699355000
N	1.306437000	-3.301380000	-1.518339000
C	-0.660834000	-3.308765000	-2.946400000
C	2.645022000	-3.574362000	0.492820000
C	0.904286000	-5.177369000	-4.722045000
O	-0.188855000	-4.913841000	-5.197359000
O	1.702153000	-6.147932000	-5.241420000
C	1.173078000	-6.842679000	-6.390536000
C	2.207213000	-7.866091000	-6.822203000
C	4.339525000	-5.448312000	-1.151332000
O	4.794482000	-5.298923000	-0.028412000
O	4.807500000	-6.402191000	-1.999283000
C	5.877408000	-7.223939000	-1.486448000
C	6.244643000	-8.222661000	-2.568563000
C	3.827820000	-3.580142000	-3.964693000
H	3.328199000	-5.680502000	-3.644449000
H	0.636174000	-2.497149000	-0.812053000
H	-0.734573000	-2.703453000	-3.854125000
H	-1.297981000	-4.182130000	-3.110350000
H	-1.033388000	-2.735725000	-2.096518000
H	1.827107000	-2.965369000	0.879664000
H	2.713837000	-4.505202000	1.062506000
H	3.592664000	-3.051298000	0.647229000
H	0.222985000	-7.310886000	-6.113038000
H	0.960618000	-6.113467000	-7.179210000
H	1.838747000	-8.421759000	-7.691452000
H	3.148818000	-7.380466000	-7.098922000
H	2.411207000	-8.580385000	-6.017942000
H	5.535704000	-7.717250000	-0.570728000
H	6.720875000	-6.579927000	-1.216464000
H	7.051683000	-8.873631000	-2.214870000
H	6.587252000	-7.712210000	-3.474701000
H	5.386005000	-8.849573000	-2.830105000
H	3.463587000	-2.632579000	-3.545392000
C	5.330250000	-3.689179000	-3.668831000
H	5.735319000	-4.648933000	-4.009011000
H	5.868118000	-2.892846000	-4.197295000
H	5.554503000	-3.582927000	-2.603127000
C	3.594561000	-3.554847000	-5.481899000
H	3.887240000	-4.503516000	-5.945736000
H	2.550285000	-3.356542000	-5.741602000
H	4.200404000	-2.760356000	-5.934180000

^{4}IM

Fe	-0.003895000	0.001385000	0.000951000
N	-0.016229000	-0.058269000	2.017442000
C	1.076221000	-0.054212000	2.848942000
C	0.650353000	-0.189413000	4.220003000
C	-0.707014000	-0.295182000	4.202869000
C	-1.115850000	-0.223332000	2.821925000
H	1.318806000	-0.210352000	5.071325000
H	-1.385053000	-0.420326000	5.037489000
C	-2.430424000	-0.329946000	2.390190000
H	-3.197480000	-0.454572000	3.147817000
N	-2.015100000	-0.141978000	-0.019074000
C	-2.837123000	-0.293116000	1.064927000
C	-4.212144000	-0.384108000	0.632067000
C	-4.204869000	-0.275457000	-0.723691000
C	-2.825655000	-0.117222000	-1.122047000
H	-5.057930000	-0.509779000	1.296030000
H	-5.043507000	-0.292687000	-1.408065000
C	-2.406067000	0.061380000	-2.431455000

H	-3.165787000	0.064640000	-3.206411000
N	0.003587000	0.264927000	-1.996656000
C	-1.088281000	0.241731000	-2.826634000
C	-0.667222000	0.413436000	-4.195146000
C	0.689113000	0.529724000	-4.180213000
C	1.102294000	0.426505000	-2.802447000
H	-1.337298000	0.433807000	-5.045141000
H	1.364890000	0.664152000	-5.015185000
C	2.423010000	0.454691000	-2.377543000
H	3.188972000	0.602429000	-3.132157000
N	2.017227000	0.123280000	0.022232000
C	2.837528000	0.317726000	-1.060602000
C	4.210115000	0.405643000	-0.627394000
C	4.204644000	0.285018000	0.729839000
C	2.828239000	0.124481000	1.129205000
H	5.053275000	0.560969000	-1.288588000
H	5.042403000	0.319941000	1.414809000
C	2.401489000	0.035686000	2.446596000
H	3.160960000	0.043778000	3.221963000
O	0.054841000	-1.765391000	-0.128644000
S	-0.141727000	2.324951000	0.075600000
H	-0.155984000	2.471626000	1.419041000
C	2.977839000	-3.951628000	0.839301000
C	4.188541000	-4.637329000	0.820001000
C	5.011642000	-4.613322000	-0.435009000
C	4.150349000	-4.445511000	-1.652485000
C	2.943282000	-3.762836000	-1.528856000
N	2.451862000	-3.429425000	-0.303508000
C	2.055670000	-3.375525000	-2.687101000
C	2.123169000	-3.758839000	2.068867000
C	4.605013000	-4.964543000	-2.962338000
O	4.176981000	-4.630960000	-4.054560000
O	5.567759000	-5.912367000	-2.819260000
C	6.059520000	-6.495069000	-4.045653000
C	7.090829000	-7.543763000	-3.672185000
C	4.686536000	-5.344299000	2.022021000
O	4.303834000	-5.169798000	3.166808000
O	5.633156000	-6.266180000	1.706224000
C	6.163686000	-7.023244000	2.815559000
C	7.142650000	-8.036223000	2.251098000
C	6.052214000	-3.381315000	-0.355222000
H	5.614858000	-5.519088000	-0.514368000
H	0.961614000	-2.159660000	-0.178302000
H	2.564112000	-2.684251000	-3.364785000
H	1.791242000	-4.250719000	-3.288441000
H	1.146744000	-2.909745000	-2.303071000
H	1.190888000	-3.267444000	1.786000000
H	1.901571000	-4.717033000	2.548085000
H	2.640532000	-3.155651000	2.820221000
H	5.215708000	-6.925159000	-4.594606000
H	6.485431000	-5.701105000	-4.668098000
H	7.486877000	-8.013706000	-4.579038000
H	7.926621000	-7.096338000	-3.124171000
H	6.646357000	-8.324083000	-3.045959000
H	5.333643000	-7.502733000	3.343825000
H	6.643843000	-6.332299000	3.516647000
H	7.566642000	-8.635004000	3.064572000
H	7.964838000	-7.539131000	1.725769000
H	6.643911000	-8.712443000	1.549318000
H	5.431397000	-2.479376000	-0.276609000
C	6.953804000	-3.456992000	0.884965000
H	7.545237000	-4.379403000	0.894523000
H	7.652733000	-2.612132000	0.878259000
H	6.388272000	-3.403297000	1.819921000
C	6.917638000	-3.264683000	-1.617789000
H	7.509629000	-4.171906000	-1.783055000
H	6.325268000	-3.074208000	-2.517543000
H	7.615740000	-2.426576000	-1.504518000

^{2}IM

Fe	-0.004620000	0.002034000	0.001186000
N	-0.016869000	-0.057910000	2.017631000
C	1.075658000	-0.054458000	2.849070000
C	0.649808000	-0.189705000	4.220134000
C	-0.707594000	-0.294970000	4.203059000
C	-1.116477000	-0.222718000	2.822149000
H	1.318311000	-0.211081000	5.071406000
H	-1.385636000	-0.420030000	5.037691000
C	-2.431097000	-0.328772000	2.390431000
H	-3.198199000	-0.453288000	3.148025000
N	-2.015661000	-0.140363000	-0.018818000
C	-2.837756000	-0.291424000	1.065185000
C	-4.212815000	-0.381542000	0.632335000
C	-4.205547000	-0.272350000	-0.723384000
C	-2.826295000	-0.114709000	-1.121765000
H	-5.058642000	-0.507007000	1.296284000
H	-5.044231000	-0.288851000	-1.407720000
C	-2.406745000	0.064116000	-2.431134000
H	-3.166542000	0.068136000	-3.206010000
N	0.003037000	0.265795000	-1.996432000
C	-1.088867000	0.243674000	-2.826360000
C	-0.667751000	0.415364000	-4.194859000
C	0.688679000	0.530501000	-4.179974000
C	1.101845000	0.426647000	-2.802245000
H	-1.337855000	0.436473000	-5.044813000
H	1.364527000	0.664546000	-5.014950000
C	2.422603000	0.453779000	-2.377419000
H	3.188630000	0.601012000	-3.132066000
N	2.016743000	0.122613000	0.022365000

C	2.837102000	0.316521000	-1.060489000
C	4.209757000	0.403694000	-0.627328000
C	4.204247000	0.283179000	0.729920000
C	2.827756000	0.123398000	1.129303000
H	5.052978000	0.558550000	-1.288553000
H	5.042030000	0.317681000	1.414880000
C	2.400956000	0.034778000	2.446704000
H	3.160433000	0.042449000	3.222070000
O	0.054379000	-1.765030000	-0.128419000
S	-0.140036000	2.325734000	0.076071000
H	-0.154223000	2.472224000	1.419533000
C	2.977428000	-3.953519000	0.838861000
C	4.188873000	-4.637876000	0.819837000
C	5.011888000	-4.613734000	-0.435263000
C	4.150445000	-4.446926000	-1.652832000
C	2.942527000	-3.765562000	-1.529305000
N	2.450274000	-3.433016000	-0.304199000
C	2.054815000	-3.379074000	-2.687727000
C	2.122821000	-3.760677000	2.068472000
C	4.605779000	-4.965606000	-2.962615000
O	4.178168000	-4.631845000	-4.054951000
O	5.568959000	-5.912946000	-2.819326000
C	6.061622000	-6.495078000	-4.045640000
C	7.092950000	-7.543657000	-3.671889000
C	4.687505000	-5.344021000	2.022105000
O	4.304983000	-5.169102000	3.166884000
O	5.634214000	-6.265862000	1.706504000
C	6.165124000	-7.022367000	2.816041000
C	7.143459000	-8.036022000	2.251699000
C	6.051691000	-3.381162000	-0.355965000
H	5.615681000	-5.519166000	-0.514354000
H	0.960594000	-2.159933000	-0.178587000
H	2.562267000	-2.685914000	-3.364266000
H	1.792624000	-4.254093000	-3.290252000
H	1.144750000	-2.915466000	-2.303734000
H	1.189722000	-3.271030000	1.785256000
H	1.902905000	-4.718550000	2.549071000
H	2.639479000	-3.155613000	2.818830000
H	5.218238000	-6.925138000	-4.595266000
H	6.487744000	-5.700780000	-4.667516000
H	7.489713000	-8.013173000	-4.578648000
H	7.928267000	-7.096204000	-3.123172000
H	6.648269000	-8.324303000	-3.046223000
H	5.335222000	-7.501210000	3.345102000
H	6.645916000	-6.331102000	3.516386000
H	7.567818000	-8.634311000	3.065341000
H	7.965441000	-7.539523000	1.725481000
H	6.644103000	-8.712624000	1.550726000
H	5.430439000	-2.479483000	-0.277566000
C	6.953324000	-3.455988000	0.884320000
H	7.544875000	-4.378311000	0.894436000
H	7.652167000	-2.611064000	0.877213000
H	6.387667000	-3.401847000	1.819174000
C	6.917083000	-3.264304000	-1.618417000
H	7.509873000	-4.171042000	-1.783328000
H	6.324792000	-3.074628000	-2.518357000
H	7.614486000	-2.425613000	-1.505409000

^{4}**PC**

Fe	-0.005223000	0.005850000	-0.003206000
N	-0.018166000	0.069408000	2.013280000
C	1.073698000	0.119687000	2.844137000
C	0.645545000	0.081772000	4.220354000
C	-0.712934000	-0.013063000	4.208729000
C	-1.120540000	-0.029367000	2.825972000
H	1.313069000	0.112959000	5.072098000
H	-1.392472000	-0.074943000	5.049197000
C	-2.436522000	-0.143467000	2.400699000
H	-3.204984000	-0.210872000	3.164127000
N	-2.020064000	-0.097416000	-0.015167000
C	-2.843725000	-0.174581000	1.075354000
C	-4.220524000	-0.262907000	0.647947000
C	-4.212391000	-0.226957000	-0.711742000
C	-2.830782000	-0.117342000	-1.117825000
H	-5.067991000	-0.336439000	1.317563000
H	-5.051805000	-0.264939000	-1.394315000
C	-2.408198000	-0.023655000	-2.435129000
H	-3.168205000	-0.048332000	-3.209431000
N	0.006475000	0.147894000	-2.012198000
C	-1.086935000	0.101204000	-2.839498000
C	-0.663471000	0.179115000	-4.215729000
C	0.695324000	0.262582000	-4.207693000
C	1.107546000	0.233992000	-2.826228000
H	-1.334049000	0.164225000	-5.065444000
H	1.373171000	0.329721000	-5.049081000
C	2.428924000	0.260027000	-2.402976000
H	3.197676000	0.343985000	-3.164503000
N	2.016363000	0.091427000	0.012884000
C	2.840680000	0.199243000	-1.079706000
C	4.214551000	0.291961000	-0.651711000
C	4.206963000	0.266596000	0.710076000
C	2.827965000	0.156491000	1.118157000
H	5.061165000	0.383269000	-1.320238000
H	5.046729000	0.333197000	1.390106000
C	2.400212000	0.165447000	2.437758000
H	3.159822000	0.217646000	3.211284000
O	0.010310000	-1.769332000	-0.028672000
S	-0.103438000	2.331285000	-0.070171000
H	-0.173463000	2.558128000	1.260295000

C	2.618959000	-4.085679000	1.416151000
C	3.721138000	-4.956546000	1.591594000
C	4.438955000	-5.338183000	0.457244000
C	4.072629000	-4.887183000	-0.811670000
C	2.954504000	-4.025154000	-0.910216000
N	2.287076000	-3.641323000	0.191662000
C	2.418190000	-3.490737000	-2.210883000
C	1.739247000	-3.609187000	2.540421000
C	4.847666000	-5.311740000	-2.012948000
O	4.603916000	-4.992397000	-3.160789000
O	5.889458000	-6.114552000	-1.685970000
C	6.689450000	-6.587938000	-2.793673000
C	7.771392000	-7.486309000	-2.224240000
C	4.117525000	-5.465203000	2.936348000
O	3.531485000	-5.242448000	3.978271000
O	5.237223000	-6.226969000	2.878993000
C	5.683079000	-6.784392000	4.136567000
C	6.931798000	-7.599488000	3.857117000
C	7.176061000	-2.581163000	0.875849000
H	5.287139000	-6.000506000	0.562090000
H	0.895698000	-2.202610000	0.034948000
H	3.153863000	-2.851624000	-2.709056000
H	2.204300000	-4.304429000	-2.909601000
H	1.509725000	-2.918765000	-2.017827000
H	0.947439000	-2.977144000	2.136586000
H	1.300215000	-4.456946000	3.074875000
H	2.313648000	-3.051019000	3.285825000
H	6.038251000	-7.119849000	-3.494360000
H	7.106145000	-5.723519000	-3.320064000
H	8.399901000	-7.868023000	-3.036199000
H	8.409808000	-6.937219000	-1.524473000
H	7.334607000	-8.340521000	-1.696764000
H	4.876733000	-7.395541000	4.554354000
H	5.873499000	-5.964795000	4.836402000
H	7.299453000	-8.043535000	4.788617000
H	7.725157000	-6.971310000	3.439129000
H	6.722253000	-8.408503000	3.149811000
H	6.240023000	-2.053901000	0.704926000
C	7.472431000	-3.079247000	2.252255000
H	7.071426000	-4.095754000	2.422441000
H	8.554835000	-3.139980000	2.434864000
H	7.034951000	-2.434778000	3.023979000
C	7.930134000	-3.087742000	-0.309422000
H	7.804834000	-2.436279000	-1.182521000
H	9.005778000	-3.170375000	-0.098793000
H	7.594287000	-4.096138000	-0.615241000

^{2}PC

Fe	-0.005223000	0.005850000	-0.003206000
N	-0.018166000	0.069408000	2.013280000
C	1.073698000	0.119687000	2.844137000
C	0.645545000	0.081772000	4.220354000
C	-0.712934000	-0.013063000	4.208729000
C	-1.120540000	-0.029367000	2.825972000
H	1.313069000	0.112959000	5.072098000
H	-1.392472000	-0.074943000	5.049197000
C	-2.436522000	-0.143467000	2.400699000
H	-3.204984000	-0.210872000	3.164127000
N	-2.020064000	-0.097416000	-0.015167000
C	-2.843725000	-0.174581000	1.075354000
C	-4.220524000	-0.262907000	0.647947000
C	-4.212391000	-0.226957000	-0.711742000
C	-2.830782000	-0.117342000	-1.117825000
H	-5.067991000	-0.336439000	1.317563000
H	-5.051805000	-0.264939000	-1.394315000
C	-2.408198000	-0.023655000	-2.435129000
H	-3.168205000	-0.048332000	-3.209431000
N	0.006475000	0.147894000	-2.012198000
C	-1.086935000	0.101204000	-2.839498000
C	-0.663471000	0.179115000	-4.215729000
C	0.695324000	0.262582000	-4.207693000
C	1.107546000	0.233992000	-2.826228000
H	-1.334049000	0.164225000	-5.065444000
H	1.373171000	0.329721000	-5.049081000
C	2.428924000	0.260027000	-2.402976000
H	3.197676000	0.343985000	-3.164503000
N	2.016363000	0.091427000	0.012884000
C	2.840680000	0.199243000	-1.079706000
C	4.214551000	0.291961000	-0.651711000
C	4.206963000	0.266596000	0.710076000
C	2.827965000	0.156491000	1.118157000
H	5.061165000	0.383269000	-1.320238000
H	5.046729000	0.333197000	1.390106000
C	2.400212000	0.165447000	2.437758000
H	3.159822000	0.217646000	3.211284000
O	0.010310000	-1.769332000	-0.028672000
S	-0.103438000	2.331285000	-0.070171000
H	-0.173463000	2.558128000	1.260295000
C	2.618959000	-4.085679000	1.416151000
C	3.721138000	-4.956546000	1.591594000
C	4.438955000	-5.338183000	0.457244000
C	4.072629000	-4.887183000	-0.811670000
C	2.954504000	-4.025154000	-0.910216000
N	2.287076000	-3.641323000	0.191662000
C	2.418190000	-3.490737000	-2.210883000
C	1.739247000	-3.609187000	2.540421000
C	4.847666000	-5.311740000	-2.012948000
O	4.603916000	-4.992397000	-3.160789000
O	5.889458000	-6.114552000	-1.685970000

C	6.689450000	-6.587938000	-2.793673000
C	7.771392000	-7.486309000	-2.224240000
C	4.117525000	-5.465203000	2.936348000
O	3.531485000	-5.242448000	3.978271000
O	5.237223000	-6.226969000	2.878993000
C	5.683079000	-6.784392000	4.136567000
C	6.931798000	-7.599488000	3.857117000
C	7.176061000	-2.581163000	0.875849000
H	5.287139000	-6.000506000	0.562090000
H	0.895698000	-2.202610000	0.034948000
H	3.153863000	-2.851624000	-2.709056000
H	2.204300000	-4.304429000	-2.909601000
H	1.509725000	-2.918765000	-2.017827000
H	0.947439000	-2.977144000	2.136586000
H	1.300215000	-4.456946000	3.074875000
H	2.313648000	-3.051019000	3.285825000
H	6.038251000	-7.119849000	-3.494360000
H	7.106145000	-5.723519000	-3.320064000
H	8.399901000	-7.868023000	-3.036199000
H	8.409808000	-6.937219000	-1.524473000
H	7.334607000	-8.340521000	-1.696764000
H	4.876733000	-7.395541000	4.554354000
H	5.873499000	-5.964795000	4.836402000
H	7.299453000	-8.043535000	4.788617000
H	7.725157000	-6.971310000	3.439129000
H	6.722253000	-8.408503000	3.149811000
H	6.240023000	-2.053901000	0.704926000
C	7.472431000	-3.079247000	2.252255000
H	7.071426000	-4.095754000	2.422441000
H	8.554835000	-3.139980000	2.434864000
H	7.034951000	-2.434778000	3.023979000
C	7.930134000	-3.087742000	-0.309422000
H	7.804834000	-2.436279000	-1.182521000
H	9.005778000	-3.170375000	-0.098793000
H	7.594287000	-4.096138000	-0.615241000
